# Nearest Neighbor Decoding and Pilot-Aided Channel Estimation for Fading Channels †

**DOI:** 10.3390/e22090971

**Published:** 2020-08-31

**Authors:** A. Taufiq Asyhari, Tobias Koch, Albert Guillén i Fàbregas

**Affiliations:** 1 School of Computing and Digital Technology, Birmingham City University, Millennium Point, Birmingham B4 7XG, UK; 2 Signal Theory and Communications Department, Universidad Carlos III de Madrid, 28911 Leganés, Spain; koch@tsc.uc3m.es; 3 Gregorio Marañón Health Research Institute, 28007 Madrid, Spain; 4 Department of Information and Communication Technologies, Universitat Pompeu Fabra, 08018 Barcelona, Spain; guillen@ieee.org; 5 Institució Catalana de Recerca i Estudis Avançats (ICREA), 08010 Barcelona, Spain; 6 Department of Engineering, University of Cambridge, Cambridge CB2 1PZ, UK

**Keywords:** achievable rates, fading, high signal-to-noise ratio (SNR), mismatched decoding, multiple-access channels, multiple antennas, nearest neighbor decoding, noncoherent, pilot-aided channel estimation

## Abstract

We study the information rates of noncoherent, stationary, Gaussian, and multiple-input multiple-output (MIMO) flat-fading channels that are achievable with nearest neighbor decoding and pilot-aided channel estimation. In particular, we investigate the behavior of these achievable rates in the limit as the signal-to-noise ratio (SNR) tends to infinity by analyzing the capacity pre-log, which is defined as the limiting ratio of the capacity to the logarithm of the SNR as the SNR tends to infinity. We demonstrate that a scheme estimating the channel using pilot symbols and detecting the message using nearest neighbor decoding (while assuming that the channel estimation is perfect) essentially achieves the capacity pre-log of noncoherent multiple-input single-output flat-fading channels, and it essentially achieves the best so far known lower bound on the capacity pre-log of noncoherent MIMO flat-fading channels. Extending the analysis to fading multiple-access channels reveals interesting relationships between the number of antennas and Doppler bandwidth in the comparative performance of joint transmission and time division multiple-access.

## 1. Introduction

The capacity of coherent multiple-input multiple-output (MIMO) channels increases with the signal-to-noise ratio (SNR) as $\min(n_{t},n_{r})\log\mathrm{SNR}$, where $n_{t}$ and $n_{r}$ are the number of transmit and receive antennas, respectively, and SNR denotes the SNR per receive antenna [1,2]. The growth factor $\min(n_{t},n_{r})$ is sometimes referred to as the capacity pre-log [3] or spatial multiplexing gain [4,5,6]. This capacity growth can be achieved using a nearest neighbor decoder which selects the codeword that is closest (in Euclidean distance) to the channel output. In fact, for coherent fading channels with additive Gaussian noise, this decoder is the maximum-likelihood decoder and is therefore optimal in the sense that it minimizes the error probability (see [7] and references therein). The coherent channel model assumes that there is a genie that provides the exact fading coefficients to the decoder, an assumption that is difficult to achieve in practice. In this paper, we replace the role of the genie by a scheme that estimates the fading coefficients via pilot symbols. This can be viewed as a particular coding strategy over a noncoherent fading channel, i.e., a channel where both communication ends do not have access to fading coefficients but may be aware of the fading statistics. Please note that with imperfect fading estimation, the nearest neighbor decoder that treats the fading estimate as if it were perfect is not necessarily optimal. Nevertheless, we show that in some relevant cases, nearest neighbor decoding with pilot-aided channel estimation achieves the capacity pre-log of noncoherent fading channels. (For noncoherent channels, the *capacity pre-log* is defined as the limiting ratio of the capacity to the logarithm of the SNR as the SNR tends to infinity.)

The capacity of noncoherent fading channels has been studied in several works. Building upon [8], Hassibi and Hochwald [9] studied the capacity of the block-fading channel and used pilot symbols (also known as training symbols) to obtain reasonably accurate fading estimates. Jindal and Lozano [10] provided tools for a unified treatment of pilot-based channel estimation in both block and stationary fading channels with bandlimited power spectral densities. In these works, lower bounds on the channel capacity were obtained. Lapidoth [3] studied a single-input single-output (SISO) fading channel for more general stationary fading processes and showed that depending on the predictability of the fading process, the capacity growth in SNR can be, *inter alia*, logarithmic or double logarithmic. The extension of [3] to multiple-input single-output (MISO) fading channels can be found in [11]. A lower bound on the capacity of stationary MIMO fading channels was derived by Etkin and Tse in [12]. With a view to next-generation (5G and beyond) communication networks, there has been an interest in capacity analyses of noncoherent massive MIMO channels with the vast majority of attempts focusing on the block-fading model [13,14,15].

Lapidoth and Shamai [16] and Weingarten et al. [17] studied noncoherent stationary fading channels from a mismatched-decoding perspective. In particular, they studied the achievable rates of Gaussian codebooks and nearest neighbor decoding. In both works, it is assumed that there is a genie that provides imperfect estimates of the fading coefficients.

In this work, we add the estimation of the fading coefficients to the analysis. In particular, we study a communication system where the transmitter emits pilot symbols at regular intervals, and where the receiver separately performs *channel estimation* and *data detection*. More precisely, based on the channel outputs corresponding to pilot transmissions, the channel estimator produces estimates of the fading coefficients for the remaining time instants using a linear minimum mean-squared error (LMMSE) interpolator. Using these estimates, the data detector employs a nearest neighbor decoder that detects the transmitted message. We study the achievable rates of this communication scheme at high SNR. In particular, we study the pre-log for fading processes with bandlimited power spectral densities. (The *pre-log* is defined as the limiting ratio of the achievable rate to the logarithm of the SNR as the SNR tends to infinity.)

For SISO fading channels, using simplifying arguments, Lozano [18] and Jindal and Lozano [10] showed that this scheme achieves the capacity pre-log. In particular, they express the achievable rates of this scheme in terms of the capacity of a fading channel whose SNR is reduced due to the imperfect channel estimation; (cf. [10], Equation (17)). Their expression ([10], Equation (21)) for the estimation error is based on the assumption that channel estimation is performed using infinitely many pilot symbols. However, obtaining ([10], Equation (17)) from the provided references is not straightforward, since it requires a limiting argument where both the codeword length and the number of pilot symbols tend to infinity in a controlled manner. The analysis is further complicated by the fact that for a given number of pilot symbols, the estimation error of the LMMSE interpolator is not stationary but cyclo-stationary, and it becomes stationary only as the number of pilot symbols tends to infinity. In this paper, we prove this result without any simplifying assumptions and extend it to MIMO fading channels. We show that the maximum rate pre-log with nearest neighbor decoding and pilot-aided channel estimation is given by the capacity pre-log of the coherent fading channel times the fraction of time used for the transmission of data. Hence, the loss with respect to the coherent case is solely due to the transmission of pilots used to obtain accurate fading estimates. If the inverse of twice the bandwidth of the fading process is an integer, then for MISO channels, the above scheme achieves the capacity pre-log derived by Koch and Lapidoth [11]. For MIMO channels, the above scheme achieves the best so far known lower bound on the capacity pre-log obtained in [12]. The proof steps followed in this paper apply also to other pilot-assisted communication strategies and can be mimicked to perform rigorous analyses of their achievable rates; see, e.g., [19,20,21,22].

The rest of the paper is organized as follows. Section 2 describes the channel model and introduces our transmission scheme along with nearest neighbor decoding and pilots for channel estimation. Section 3 defines the pre-log and presents the main result. Section 4 extends the use of our scheme to a fading multiple-access channel (MAC). Section 5 and Section 6 provide the proofs of our main results. Section 7 summarizes the results and concludes the paper.

## 2. System Model and Transmission Scheme

We consider a discrete-time MIMO flat-fading channel with $n_{t}$ transmit antennas and $n_{r}$ receive antennas. Thus, the channel output at time instant k∈ℤ (where ℤ denotes the set of integers) is the complex-valued $n_{r}$-dimensional random vector given by

(1) $$Y_{k}=\sqrt{\frac{\mathrm{SNR}}{n_{t}}}H_{k}x_{k}+Z_{k}.$$

Here $x_{k}{\in \mathbb{C}}^{n_{t}}$ denotes the time-*k* channel input vector (with ℂ denoting the set of complex numbers), $H_{k}$ denotes the $\left(n_{r}\times n_{t}\right)$-dimensional random fading matrix at time *k*, and $Z_{k}$ denotes the $n_{r}$-variate random additive noise vector at time *k*.

The noise process $\left\{Z_{k},\;k\in \mathbb{Z}\right\}$ is a sequence of independent and identically distributed (i.i.d.) complex-Gaussian random vectors with zero mean and covariance matrix $I_{n_{r}}$, where $I_{n_{r}}$ is the $n_{r}\times n_{r}$ identity matrix. SNR denotes the average SNR for each received antenna. The fading process $\left\{H_{k},\;k\in \mathbb{Z}\right\}$ is stationary, ergodic, and complex-Gaussian. We assume that the $n_{r}\cdot n_{t}$ processes $\left\{H_{k}\left(r,t\right),\;k\in \mathbb{Z}\right\}$, $r=1,\ldots ,n_{r}$, $t=1,\ldots ,n_{t}$ are independent and have the same law, with each process having zero mean, unit variance, and power spectral density $f_{H}\left(\lambda \right)$, $-\frac{1}{2}\leq \lambda \leq \frac{1}{2}$. The assumption that the fading processes are independent is realistic for data transmission over a rich uniform scattering environment when both transmit and receive antennas have sufficient separation to ensure independent signal paths that translate into spatially-independent fading coefficients. The power spectral density $f_{H}\left(\cdot \right)$ is a nonnegative (measurable) function satisfying

(2) $$E\left[H_{k+m}\left(r,t\right)H_{k}^{*}\left(r,t\right)\right]=\int_{-1/2}^{1/2}e^{i2\pi m\lambda }f_{H}\left(\lambda \right)d\lambda$$

where ${\left(\cdot \right)}^{*}$ denotes complex conjugation, and where $i≜\sqrt{-1}$. We assume that $f_{H}\left(\cdot \right)$ has bandwidth $\lambda_{D}<1/2$, i.e., $\lambda_{D}$ is the smallest value such that $f_{H}\left(\lambda \right)=0$ for almost every $|\lambda |>\lambda_{D}$. We finally assume that the fading process $\left\{H_{k},\;k\in \mathbb{Z}\right\}$ and the noise process $\left\{Z_{k},\;k\in \mathbb{Z}\right\}$ are independent and that their joint law does not depend on $\left\{x_{k},\;k\in \mathbb{Z}\right\}$.

The transmission involves both codewords and pilots. The former conveys the message to be transmitted, and the latter are used to facilitate the estimation of the fading coefficients at the receiver. We denote a codeword conveying a message *m*, m∈M at rate *R* (where $M=\left\{1,\cdots ,\lfloor e^{nR}\rfloor \right\}$ is the set of possible messages, and where ⌊b⌋ denotes the largest integer smaller than or equal to *b*) by the length-*n* sequence of input vectors ${\bar{x}}_{1}\left(m\right),\cdots ,{\bar{x}}_{n}\left(m\right)$. The codeword is selected from the codebook C, which is drawn i.i.d. from an $n_{t}$-variate complex-Gaussian distribution with zero mean and identity covariance matrix, so

(3) $$\frac{1}{n}\sum_{k=1}^{n}E\left[{∥{\bar{X}}_{k}\left(m\right)∥}^{2}\right]=n_{t},\;\;m\in M$$

where ∥·∥ denotes the Euclidean norm.

To estimate the fading matrix, we transmit orthogonal pilot vectors. The pilot vector $p_{t}\in {\mathbb{C}}^{n_{t}}$ used to estimate the fading coefficients corresponding to the *t*-th transmit antenna is given by $p_{t}\left(t\right)=1$ and $p_{t}\left(t^{\prime }\right)=0$ for $t^{\prime }\neq t$. For example, the first pilot vector is $p_{1}={\left(1,0,\cdots ,0\right)}^{T}$, where ${\left(\cdot \right)}^{T}$ denotes the transpose. To estimate the whole fading matrix, we thus need to send the $n_{t}$ pilot vectors $p_{1},\ldots ,p_{n_{t}}$.

**Figure 1:**
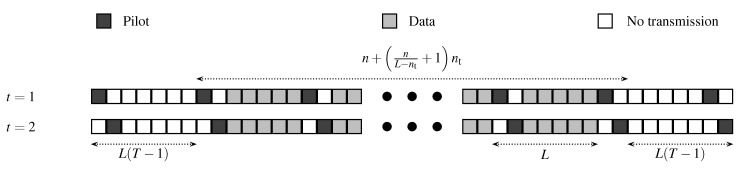
Structure of pilot and data transmission for $n_{t}=2$, L=7, and T=2.

The transmission scheme is as follows. Every *L* time instants (for some L∈ℕ, where ℕ is the set of positive integers), we transmit the $n_{t}$ pilot vectors $p_{1},\ldots ,p_{n_{t}}$. Each codeword is then split up into blocks of $L-n_{t}$ data vectors, which will be transmitted after the $n_{t}$ pilot vectors. The process of transmitting $L-n_{t}$ data vectors and $n_{t}$ pilot vectors continues until all *n* data vectors are completed. Herein we assume that *n* is an integer multiple of $L-n_{t}$. (If *n* is not an integer multiple of $L-n_{t}$, then the last $L-n_{t}$ instants are not fully used by data vectors and include therefore time instants where we do not transmit anything. The thereby incurred loss in information rate vanishes as *n* tends to infinity.) Prior to transmitting the first data block, and after transmitting the last data block, we introduce a guard period of L(T−1) time instants (for some T∈ℕ), where we transmit every *L* time instants the $n_{t}$ pilot vectors $p_{1},\ldots ,p_{n_{t}}$, but we do not transmit data vectors in between. The guard period ensures that at every time instant, we can employ a channel estimator that bases its estimation on the channel outputs corresponding to the *T* preceding and the *T* subsequent pilot transmissions. This facilitates the analysis and, asymptotically, does not incur any loss in terms of achievable rates. The above transmission scheme is illustrated in Figure 1. The channel estimator is described in the following.

Please note that the total blocklength of the above transmission scheme (comprising data vectors, pilot vectors, and guard period) is given by

(4) $$n^{\prime }=n_{p}+n+n_{g}$$

where $n_{p}$ denotes the number of channel uses reserved for pilot vectors, and where $n_{g}$ denotes the number of channel uses during the silent guard period, i.e.,

(5) $$\begin{array}{cc} n_{p} & =\left(\frac{n}{L-n_{t}}+1+2\left(T-1\right)\right)n_{t} \end{array}$$

(6) $$\begin{array}{cc} n_{g} & =2\left(L-n_{t}\right)\left(T-1\right). \end{array}$$

We now turn to the decoder. Let D denote the set of integers reserved for the transmission of data vectors, and let P denote the set of integers reserved for the transmission of pilot symbols. The decoder consists of two parts: a *channel estimator* and a *data detector*. To estimate the fading coefficient at a given time instant, the channel estimator considers the channel output vectors $Y_{k^{\prime }}$, $k^{\prime }\in P$ corresponding to the *T* preceding and *T* subsequent pilot transmissions and estimates $H_{k}\left(r,t\right)$ using a linear interpolator. The estimate ${\hat{H}}_{k}^{\left(T\right)}\left(r,t\right)$ of the fading coefficient $H_{k}\left(r,t\right)$ is thus given by

(7) $${\hat{H}}_{k}^{\left(T\right)}\left(r,t\right)=\sum_{\begin{array}{c} k^{\prime }=k-TL:\\ k^{\prime }\in P \end{array}}^{k+TL}a_{k^{\prime }}\left(r,t\right)Y_{k^{\prime }}\left(r\right)$$

where the coefficients $a_{k^{\prime }}\left(r,t\right)$ are chosen in order to minimize the mean-squared error. (It has been shown in [23] that for the linear interpolator in (7), only the observations when pilots are transmitted, i.e., $Y_{k^{\prime }},\;k^{\prime }\in P$ are relevant for fading estimation.) In general, these coefficients depend on *k* and *T*. However, for the sake of compactness, we do not reflect this dependence in the notation.

Please note that since the pilot vectors transmit only from one antenna, the fading coefficients corresponding to all transmit and receive antennas (r,t) can be observed. Furthermore, please note that since the fading processes $\left\{H_{k}\left(r,t\right),\;k\in \mathbb{Z}\right\}$, $r=1,\ldots ,n_{r}$, $t=1,\ldots ,n_{t}$ are independent, estimating $H_{k}\left(r,t\right)$ only based on $\left\{Y_{k}\left(r\right),\;k\in \mathbb{Z}\right\}$ rather than on $\left\{Y_{k},\;k\in \mathbb{Z}\right\}$ incurs no loss in optimality.

Since the time-lags between $H_{k}$, k∈D and the observations $Y_{k^{\prime }}$, $k^{\prime }\in P$ depend on *k*, it follows that the interpolation error

(8) $$E_{k}^{\left(T\right)}\left(r,t\right)≜H_{k}\left(r,t\right)-{\hat{H}}_{k}^{\left(T\right)}\left(r,t\right)$$

is not stationary but cyclo-stationary with period *L*. It can be shown that, irrespective of *r*, the variance of the interpolation error

(9) $$\epsilon_{\ell ,T}^{2}\left(r,t\right)≜E\left[{\left|H_{k}\left(r,t\right)-{\hat{H}}_{k}^{\left(T\right)}\left(r,t\right)\right|}^{2}\right]$$

tends to the following expression as *T* tends to infinity [23]:

$$\begin{array}{ccc} (10) & \epsilon_{\ell }^{2}\left(t\right) & ≜\lim_{T\to \infty }\epsilon_{\ell ,T}^{2}\left(r,t\right)\\ (11)\; & \; & =1-\int_{-1/2}^{1/2}\frac{\mathrm{SNR}|f_{L,\ell -t+1}{\left(\lambda \right)|}^{2}}{\mathrm{SNR}f_{L,0}\left(\lambda \right)+n_{t}}d\lambda \end{array}$$

where ℓ≜kmodL denotes the remainder of k/L. Here $f_{L,\ell }\left(\cdot \right)$ is given by

(12) $$f_{L,\ell }\left(\lambda \right)=\frac{1}{L}\sum_{\nu =0}^{L-1}{\bar{f}}_{H}\left(\frac{\lambda -\nu }{L}\right)e^{i2\pi \ell \frac{\lambda -\nu }{L}},\;\ell =0,\cdots ,L-1$$

and ${\bar{f}}_{H}\left(\cdot \right)$ is the periodic continuation of $f_{H}\left(\cdot \right)$, i.e., it is the periodic function of period [−1/2,1/2) that coincides with $f_{H}\left(\lambda \right)$ for −1/2≤λ≤1/2. If

(13) $$L\leq \frac{1}{2\lambda_{D}}$$

then $|f_{L,\ell }\left(\cdot \right)|$ becomes

(14) $$|f_{L,\ell }\left(\lambda \right)|=f_{L,0}\left(\lambda \right)=\frac{1}{L}f_{H}\left(\frac{\lambda }{L}\right).$$

In this case, irrespective of *ℓ* and *t*, the variance of the interpolation error is given by

(15) $$\epsilon_{\ell }^{2}\left(t\right)=\epsilon^{2}=1-\int_{-1/2}^{1/2}\frac{\mathrm{SNR}{\left[f_{H}\left(\lambda \right)\right]}^{2}}{\mathrm{SNR}f_{H}\left(\lambda \right)+Ln_{t}}d\lambda ,\;\ell =0,\cdots ,L-1,\;t=1,\cdots ,n_{t}$$

which vanishes as the SNR tends to infinity. Recall that $\lambda_{D}$ denotes the bandwidth of $f_{H}\left(\cdot \right)$. Thus, (13) implies that no aliasing occurs as we undersample the fading process *L* times. Please note that in contrast to (11), the variance in (15) is independent of the transmit antenna index *t*. See Section 5.1 for a more detailed discussion.

The channel estimator feeds the sequence of fading estimates $\left\{{\hat{H}}_{k}^{\left(T\right)},\;k\in D\right\}$ (which is composed of the matrix entries $\left\{{\hat{H}}_{k}^{\left(T\right)}\left(r,t\right),\;k\in D\right\}$) to the data detector. We shall denote its realization by $\left\{{\hat{H}}_{k}^{\left(T\right)},k\in D\right\}$. Based on the channel outputs $\left\{y_{k},\;k\in D\right\}$ and fading estimates $\left\{{\hat{H}}_{k}^{\left(T\right)},\;k\in D\right\}$, the data detector uses a nearest neighbor decoder to guess which message was transmitted. Thus, the decoder decides on the message $\hat{m}$ that satisfies

(16) $$\hat{m}=\arg\min_{m\in M}D\left(m\right)$$

where

(17) $$D\left(m\right)≜\sum_{k\in D^{\left(n^{\prime }\right)}}{∥y_{k}-\sqrt{\frac{\mathrm{SNR}}{n_{t}}}\;{\hat{H}}_{k}^{\left(T\right)}x_{k}\left(m\right)∥}^{2}.$$

On the RHS of (17), assuming that the first pilot symbol is transmitted at time k=0, we defined

(18) $$D^{\left(n^{\prime }\right)}≜\left\{0,\cdots ,n^{\prime }-1\right\}\cap D$$

as the set of time indices where data vectors corresponding to a codeword of length $n^{\prime }$ are transmitted. (For comparison, D represents the set of *all* integers that are reserved for the transmission of data vectors).

## 3. The Pre-Log

We say that a rate

(19) $$R\left(\mathrm{SNR}\right)≜\frac{\log|M|}{n}$$

is achievable if there exists a code of length *n* with |M| codewords such that the error probability tends to zero as *n* tends to infinity. In this work, we study the set of rates that are achievable with nearest neighbor decoding and pilot-aided channel estimation. We focus on the achievable rates at high SNR. In particular, we are interested in the maximum achievable pre-log, defined as

(20) $$\Pi_{R^{*}}≜\underset{\mathrm{SNR}\to \infty }{lim sup}\;\frac{R^{*}\left(\mathrm{SNR}\right)}{\log\mathrm{SNR}}$$

where $R^{*}\left(\mathrm{SNR}\right)$ is the maximum rate achievable with nearest neighbor decoding and pilot-aided channel estimation, maximized over all possible encoders.

The capacity pre-log—which is given by (20) but with $R^{*}\left(\mathrm{SNR}\right)$ replaced by the capacity C(SNR)—of SISO fading channels was computed by Lapidoth [3] as

(21) $$\Pi_{C}=\mu \left(\left\{\lambda :f_{H}\left(\lambda \right)=0\right\}\right)$$

where μ(·) denotes the Lebesgue measure on the interval [−1/2,1/2]. (The capacity is defined as the supremum of all achievable rates maximized over all possible encoders and decoders.) Koch and Lapidoth [11] extended this result to MISO fading channels and showed that if the fading processes $\left\{H_{k}\left(t\right),\;k\in \mathbb{Z}\right\}$, $t=1,\ldots ,n_{t}$ are independent and have the same law, then the capacity pre-log of MISO fading channels is equal to the capacity pre-log of the SISO fading channel with fading process $\left\{H_{k}\left(1\right),\;k\in \mathbb{Z}\right\}$. Using (21), the capacity pre-log of MISO fading channels with bandlimited power spectral densities of bandwidth $\lambda_{D}$ can be evaluated as

(22) $$\Pi_{C}=1-2\lambda_{D}.$$

Since $R^{*}\left(\mathrm{SNR}\right)\leq C\left(\mathrm{SNR}\right)$, it follows that $\Pi_{R^{*}}\leq \Pi_{C}$.

To the best of our knowledge, the capacity pre-log of MIMO fading channels is unknown. For independent fading processes $\left\{H_{k}\left(r,t\right),\;k\in \mathbb{Z}\right\}$, $t=1,\cdots ,n_{t}$, $r=1,\ldots ,n_{r}$ that have the same law, the best so far known lower bound on the MIMO pre-log is due to Etkin and Tse [12], and is given by

(23) $$\Pi_{C}\geq \min\left(n_{t},n_{r}\right)\left(1-\min\left(n_{t},n_{r}\right)\mu \left(\{\lambda :f_{H}\left(\lambda \right)>0\}\right)\right).$$

For power spectral densities that are bandlimited to $\lambda_{D}$, this becomes

(24) $$\Pi_{C}\geq \min\left(n_{t},n_{r}\right)\left(1-\min\left(n_{t},n_{r}\right)\;2\lambda_{D}\right).$$

Observe that (24) specializes to (22) for $n_{r}=1$.

It should be noted that the capacity pre-log for MISO and SISO fading channels was derived under a peak-power constraint on the channel inputs, whereas the lower bound on the capacity pre-log for MIMO fading channels was derived under an average-power constraint. Clearly, the capacity pre-log corresponding to a peak-power constraint can never be larger than the capacity pre-log corresponding to an average-power constraint. It is believed that the two pre-logs are in fact identical (see the conclusions in [3]).

In this paper, we show that a communication scheme that employs nearest neighbor decoding and pilot-aided channel estimation achieves the following pre-log.

**Theorem** **1.** *Consider the Gaussian MIMO flat-fading channel with $n_{t}$ transmit antennas and $n_{r}$ receive antennas *(1)*. Then, the transmission and decoding scheme described in Section 2 achieves*

(25) $$\Pi_{R^{*}}\geq \min\left(n_{t},n_{r}\right)\left(1-\frac{\min(n_{t},n_{r})}{L^{*}}\right)$$

where $L^{*}=\left\lfloor \frac{1}{2\lambda_{D}}\right\rfloor$.

**Proof.**  See Section 5. □

**Remark** **1.** *We derive Theorem 1 for i.i.d. Gaussian codebooks, which satisfy the average-power constraint *(3)*. Nevertheless, it can be shown that Theorem 1 continues to hold when the channel inputs satisfy a peak-power constraint. More specifically, we show in Section 5.3 that for an input distribution with power constraint $E[∥\bar{X}{∥}^{2}]\leq n_{t}$ to achieve the pre-log *(25)*, it is sufficient that its probability density function $p_{\bar{X}}\left(\cdot \right)$ satisfies*

(26) $$p_{\bar{X}}\left(\bar{x}\right)\leq \frac{K}{\pi^{n_{t}}}e^{-∥\bar{x}{∥}^{2}},\;\bar{x}\in {\mathbb{C}}^{n_{t}}$$

*for some K satisfying*

(27) $$\lim_{\mathrm{SNR}\to \infty }\;\frac{\log K}{\log\mathrm{SNR}}=0.$$

*The condition (26) is satisfied, for example, by i.i.d., truncated, Gaussian inputs, i.e., by inputs for which the $n_{t}$ elements in $\bar{X}$ are i.i.d. and*

(28) $$\begin{array}{c} p_{\bar{X}}\left(\bar{x}\right)=\left\{\begin{array}{cc} \frac{1}{\hat{K}\pi^{n_{t}}}e^{-∥\bar{x}{∥}^{2}},\; & \mathrm{if}|\bar{x}\left(t\right)|\leq 1,1\leq t\leq n_{t}\\ 0,\; & \mathrm{otherwise} \end{array}\right. \end{array}$$

*with*

(29) $$\hat{K}={\left(\int_{|\bar{x}|\leq 1}\frac{1}{\pi }e^{-|\bar{x}{|}^{2}}d\bar{x}\right)}^{n_{t}}.$$

If $1/(2\lambda_{D})$ is an integer, then (25) becomes

(30) $$\Pi_{R^{*}}\geq \min\left(n_{t},n_{r}\right)\left(1-\min\left(n_{t},n_{r}\right)\;2\lambda_{D}\right).$$

Thus, in this case nearest neighbor decoding together with pilot-aided channel estimation achieves the capacity pre-log of MISO fading channels (22) as well as the lower bound on the capacity pre-log of MIMO fading channels (24).

Suppose that both transmitter and receiver use the same number of antennas, namely, ${n_{t}}^{\prime }≜{n_{r}}^{\prime }≜\min\left(n_{t},n_{r}\right)$. Then, as the codeword length tends to infinity, we have from (4)–(6) that the fraction of time consumed for the transmission of pilots is given by

(31) $$\lim_{n\to \infty }\frac{n_{p}}{n^{\prime }}=\lim_{n\to \infty }\frac{\left(\frac{n}{L-{n_{t}}^{\prime }}+1+2\left(T-1\right)\right){n_{t}}^{\prime }}{\left(\frac{n}{L-{n_{t}}^{\prime }}+1+2\left(T-1\right)\right){n_{t}}^{\prime }+n+2\left(L-{n_{t}}^{\prime }\right)\left(T-1\right)}=\frac{{n_{t}}^{\prime }}{L}.$$

Consequently, by rewriting the pre-log (25) as

(32) $$\Pi_{R^{*}}\geq {n_{t}}^{\prime }\left(1-\frac{{n_{t}}^{\prime }}{L}\right),\;L\leq \frac{1}{2\lambda_{D}}$$

we observe that the loss compared to the capacity pre-log ${n_{t}}^{\prime }=\min\left(n_{t},n_{r}\right)$ of the coherent fading channel is given by the fraction of time used for the transmission of pilots. This implies that the nearest neighbor decoder in combination with the channel estimator described in Section 2 is optimal at high SNR in the sense that it achieves the capacity pre-log of the coherent fading channel. Moreover, the achievable pre-log in Theorem 1 is the best pre-log that can be achieved by any scheme employing ${n_{t}}^{\prime }$ pilot vectors.

To achieve the pre-log in Theorem 1, we assume that the training period *L* satisfies $L\leq \frac{1}{2\lambda_{D}}$, in which case the variance of the interpolation error (15), namely

(33) $$\epsilon^{2}=1-\int_{-1/2}^{1/2}\frac{\mathrm{SNR}{\left[f_{H}\left(\lambda \right)\right]}^{2}}{\mathrm{SNR}f_{H}\left(\lambda \right)+Ln_{t}}d\lambda \approx \frac{2\lambda_{D}Ln_{t}}{\mathrm{SNR}}$$

vanishes as the reciprocal of the SNR. The achievable pre-log is then maximized by maximizing $L\leq \frac{1}{2\lambda_{D}}$. Please note that as a criterion of “perfect side information” for nearest neighbor decoding in fading channels, Lapidoth and Shamai [16] suggested that the variance of the fading estimation error should be negligible compared to the reciprocal of the SNR. The condition $L\leq \frac{1}{2\lambda_{D}}$ can thus be viewed as a sufficient condition for obtaining “nearly perfect side information” in the sense that the variance of the interpolation error is of the same order as the reciprocal of the SNR.

Of course, one could increase the training period *L* beyond $\frac{1}{2\lambda_{D}}$. By increasing *L*, we reduce the rate loss due to the transmission of pilots as indicated in (32) at the cost of obtaining a larger fading estimation error, which in turn may reduce the reliability of the nearest neighbor decoder. To understand this trade-off better, we next briefly discuss the achievable pre-log when $L>\frac{1}{2\lambda_{D}}$. Indeed, for $L>\frac{1}{2\lambda_{D}}$, the variance of the interpolation error follows from (11) as

$$\begin{array}{ccc} (34) & \epsilon_{\ell }^{2}\left(t\right) & =1-\int_{-1/2}^{1/2}\frac{\mathrm{SNR}{\left|f_{L,\ell -t+1}\left(\lambda \right)\right|}^{2}}{\mathrm{SNR}f_{L,0}\left(\lambda \right)+n_{t}}d\lambda \\ (35) & \; & =\int_{-1/2}^{1/2}\frac{n_{t}f_{L,0}\left(\lambda \right)}{\mathrm{SNR}f_{L,0}\left(\lambda \right)+n_{t}}d\lambda +\int_{-1/2}^{1/2}\frac{\mathrm{SNR}\left({\left[f_{L,0}\left(\lambda \right)\right]}^{2}-{\left|f_{L,\ell -t+1}\left(\lambda \right)\right|}^{2}\right)}{\mathrm{SNR}f_{L,0}\left(\lambda \right)+n_{t}}d\lambda . \end{array}$$

The former integral

(36) $$\int_{-1/2}^{1/2}\frac{n_{t}f_{L,0}\left(\lambda \right)}{\mathrm{SNR}f_{L,0}\left(\lambda \right)+n_{t}}d\lambda \approx \frac{n_{t}}{\mathrm{SNR}}$$

vanishes as the reciprocal of the SNR. However, we prove in Appendix B that, as the SNR tends to infinity, the latter integral

(37) $$\int_{-1/2}^{1/2}\frac{\mathrm{SNR}\left({\left[f_{L,0}\left(\lambda \right)\right]}^{2}-{\left|f_{L,\ell -t+1}\left(\lambda \right)\right|}^{2}\right)}{\mathrm{SNR}f_{L,0}\left(\lambda \right)+n_{t}}d\lambda$$

is bounded away from zero. This implies that the interpolation error (35) does not vanish as the SNR tends to infinity, and the pre-log achievable with the scheme described in Section 2 is zero. It thus follows that the condition $L\leq \frac{1}{2\lambda_{D}}$ is necessary in order to achieve a positive pre-log.

Comparing (24) and (25) with the capacity pre-log $\min(n_{t},n_{r})$ of the coherent fading channel, we observe that, for a fading process of bandwidth $\lambda_{D}$, the penalty for not knowing the fading coefficients is roughly ${\left(\min\left(n_{t},n_{r}\right)\right)}^{2}\cdot 2\lambda_{D}$. Consequently, the lower bound (25) does not grow linearly with $\min(n_{t},n_{r})$, but it is a quadratic function of $\min(n_{t},n_{r})$ that achieves its maximum at

(38) $$\min\left(n_{t},n_{r}\right)=\frac{L^{*}}{2}.$$

This gives rise to the lower bound

(39) $$\Pi_{R^{*}}\geq \frac{L^{*}}{4}$$

which cannot be larger than $1/(8\lambda_{D})$. The same holds for the lower bound (23).

## 4. Fading Multiple-Access Channels

**Figure 2:**
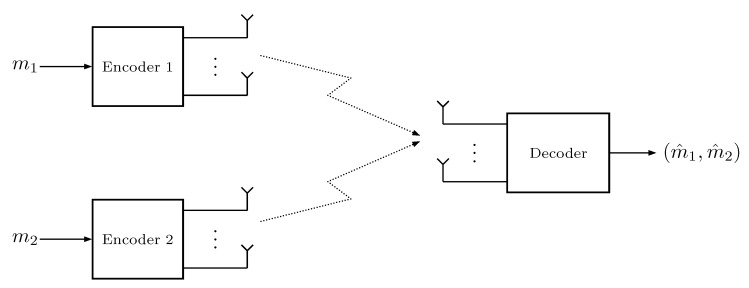
The two-user MIMO fading MAC system model.

In this section, we extend the use of nearest neighbor decoding with pilot-aided channel estimation to the fading MAC depicted in Figure 2. We are interested in the pre-log region that can be achieved with this scheme.

We consider a two-user MIMO fading MAC, where two terminals wish to communicate with a third one, and where the channels between the terminals are MIMO fading channels. Extension to more than two users is straightforward. The first user has $n_{t,1}$ antennas, the second user has $n_{t,2}$ antennas, and the receiver has $n_{r}$ antennas. The channel output at time instant k∈ℤ is a complex-valued $n_{r}$-dimensional random vector given by

(40) $$Y_{k}=\sqrt{\mathrm{SNR}}\;H_{1,k}x_{1,k}+\sqrt{\mathrm{SNR}}\;H_{2,k}x_{2,k}+Z_{k}.$$

Here $x_{s,k}\in {\mathbb{C}}^{n_{t,s}}$ denotes the time-*k* channel input vector corresponding to user *s*, s=1,2; $H_{s,k}$ denotes the $\left(n_{r}\times n_{t,s}\right)$-dimensional fading matrix at time *k* corresponding to user *s*, s=1,2; SNR denotes the average SNR for each transmit antenna; and $Z_{k}$ denotes the $n_{r}$-variate additive noise vector at time *k*. The fading processes $\left\{H_{s,k},\;k\in \mathbb{Z}\right\}$, s=1,2 are independent of each other and of the noise process $\left\{Z_{k},\;k\in \mathbb{Z}\right\}$ and have the same distribution as the fading process considered in the point-to-point channel (Section 2). The noise process $\left\{Z_{k},\;k\in \mathbb{Z}\right\}$ is a sequence of i.i.d. complex-Gaussian vectors with zero mean and covariance matrix $I_{n_{r}}$.

Both users transmit codewords and pilot symbols over the channel (40). To transmit the messages $m_{s}\in \left\{1,\cdots ,\left\lfloor e^{nR_{s}}\right\rfloor \right\}$, s=1,2 (where $m_{1}$ and $m_{2}$ are drawn independently), each user’s encoder selects a codeword of length *n* from a codebook $C_{s}$, where $C_{s}$, s=1,2 are drawn i.i.d. from an $n_{t,s}$-variate, zero-mean, complex-Gaussian distribution of covariance matrix $I_{n_{t,s}}$. Similar to the single-user case, orthogonal pilot vectors are used. The pilot vector $p_{s,t}\in {\mathbb{C}}^{n_{t,s}}$, s=1,2, $t=1,\cdots ,n_{t,s}$ used to estimate the fading coefficients from transmit antenna *t* of user *s* is given by $p_{s,t}\left(t\right)=1$ and $p_{s,t}\left(t^{\prime }\right)=0$ for $t^{\prime }\neq t$. For example, the first pilot vector of user *s* is given by ${\left(1,0,\cdots ,0\right)}^{T}$. To estimate the fading matrices $H_{1,k}$ and $H_{2,k}$, each training period requires the transmission of $\left(n_{t,1}+n_{t,2}\right)$ pilot vectors $p_{1,1},\cdots ,p_{1,n_{t,1}},p_{2,1},\cdots ,p_{2,n_{t,2}}$.

**Figure 3:**
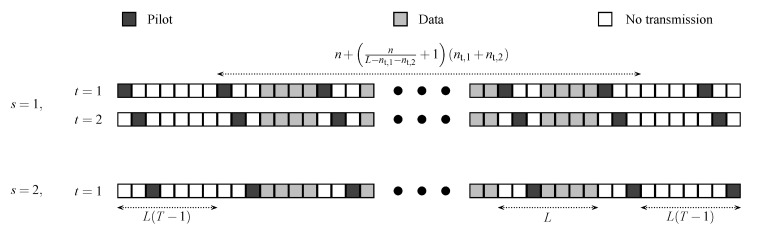
Structure of joint-transmission scheme for $n_{t,1}=2$, $n_{t,2}=1$, L=7, and T=2.

Assuming that transmission from both users is synchronized, the transmission scheme extends the point-to-point setup in Section 2 to the two-user MAC setup as illustrated in Figure 3. Every *L* time instants (for some $L\geq n_{t,1}+n_{t,2},\;L\in \mathbb{N}$), user 1 first transmits the $n_{t,1}$ pilot vectors $p_{1,1},\cdots ,p_{1,n_{t,1}}$. Once the transmission of the $n_{t,1}$ pilot vectors ends, user 2 transmits its $n_{t,2}$ pilot vectors $p_{2,1},\cdots ,p_{2,n_{t,2}}$. The codewords for both users are then split up into blocks of $\left(L-n_{t,1}-n_{t,2}\right)$ data vectors, which are transmitted simultaneously after the $\left(n_{t,1}+n_{t,2}\right)$ pilot vectors. The process of transmitting $\left(L-n_{t,1}-n_{t,2}\right)$ data vectors and $\left(n_{t,1}+n_{t,2}\right)$ pilot vectors continues until all *n* data symbols are completed. Herein we assume that *n* is an integer multiple of $\left(L-n_{t,1}-n_{t,2}\right)$. (As in the point-to-point setup, in the limit as *n* tends to infinity, this assumption is not critical in terms of achievable rates.) Prior to transmitting the first data block, and after transmitting the last data block, a guard period of L(T−1) time instants (for some T∈ℕ) is introduced for the purpose of channel estimation, where we transmit every *L* time instants the $\left(n_{t,1}+n_{t,2}\right)$ pilot vectors but we do not transmit data vectors in between. Please note that codewords from both users are *jointly* transmitted at the same time instants whereas pilots from both users do not interfere and are *separately* transmitted at different time instants. The total blocklength of this transmission scheme (comprising data vectors, pilot vectors, and guard period) is given by

(41) $$n^{\prime }=n_{p}+n+n_{g}$$

where $n_{p}$ and $n_{g}$ are

$$\begin{array}{ccc} (42) & n_{p} & =\left(\frac{n}{L-n_{t,1}-n_{t,2}}+1+2\left(T-1\right)\right)\left(n_{t,1}+n_{t,2}\right)\\ (42) & n_{g} & =2\left(L-n_{t,1}-n_{t,2}\right)\left(T-1\right). \end{array}$$

Similar to the single-user case, the receiver guesses which messages have been transmitted using a two-part decoder that consists of a channel estimator and a data detector. The channel estimator first obtains matrix-valued fading estimates $\left\{{\hat{H}}_{s,k}^{\left(T\right)},\;k\in D\right\}$, s=1,2 from the received pilots $Y_{k^{\prime }}$, $k^{\prime }\in P$ using the same linear interpolator as (7). From the received codeword $\left\{y_{k},\;k\in D\right\}$ and the channel-estimate matrices $\left\{{\hat{H}}_{s,k}^{\left(T\right)},\;k\in D\right\}$, s=1,2 (which are the realizations of $\left\{{\hat{H}}_{s,k}^{\left(T\right)},\;k\in D\right\}$, s=1,2), the decoder chooses the pair of messages $\left({\hat{m}}_{1},{\hat{m}}_{2}\right)$ that minimizes the distance metric

(44) $$\left({\hat{m}}_{1},{\hat{m}}_{2}\right)=\arg\min_{\left(m_{1},m_{2}\right)}D\left(m_{1},m_{2}\right)$$

where

(45) $$D\left(m_{1},m_{2}\right)≜\sum_{k\in D^{\left(n^{\prime }\right)}}{∥y_{k}-\sqrt{\mathrm{SNR}}\;{\hat{H}}_{1,k}^{\left(T\right)}x_{1,k}\left(m_{1}\right)-\sqrt{\mathrm{SNR}}\;{\hat{H}}_{2,k}^{\left(T\right)}x_{2,k}\left(m_{2}\right)∥}^{2}$$

and where $D^{\left(n^{\prime }\right)}$ is defined in the same way as (18). In the following, we shall refer to the above communication scheme as the *joint-transmission scheme*.

**Figure 4:**
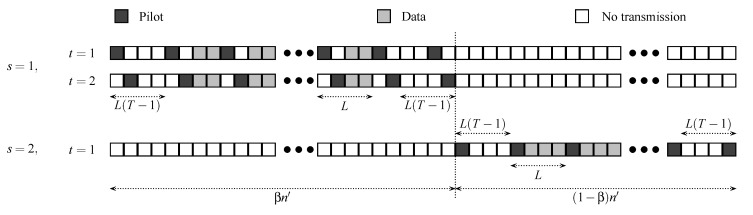
Structure of TDMA scheme for $n_{t,1}=2$, $n_{t,2}=1$, L=4, and T=2.

We shall compare the joint-transmission scheme with a time-division multiple-access (TDMA) scheme, where each user transmits its message using the transmission scheme illustrated in Figure 4. Specifically, during the first $\beta n^{\prime }$ channel uses (for some 0≤β≤1, and where $n^{\prime }$ is given in (41)), user 1 transmits its codeword according to the transmission scheme given in Section 2 (see also Figure 4), while user 2 is silent. Then, during the next $\left(1-\beta \right)n^{\prime }$ channel uses, user 2 transmits its codeword according to the same transmission scheme, while user 1 is silent. In both cases, the receiver guesses the corresponding message $m_{s}$, s=1,2 using a nearest neighbor decoder and pilot-aided channel estimation.

### 4.1. The MAC Pre-Log

Let $R_{1}^{*}\left(\mathrm{SNR}\right)$, $R_{2}^{*}\left(\mathrm{SNR}\right)$, and $R_{1+2}^{*}\left(\mathrm{SNR}\right)$ be the maximum achievable rate of user 1, the maximum achievable rate of user 2, and the maximum achievable sum-rate, respectively. The achievable-rate region is given by the set [24]

(46) $$R\left(\mathrm{SNR}\right)=\left\{\left(R_{1},R_{2}\right):R_{1}\leq R_{1}^{*}\left(\mathrm{SNR}\right),\;R_{2}\leq R_{2}^{*}\left(\mathrm{SNR}\right),\;R_{1}+R_{2}\leq R_{1+2}^{*}\left(\mathrm{SNR}\right)\right\}.$$

We are interested in the pre-logs of all rate pairs $\left(R_{1}\left(\mathrm{SNR}\right),R_{2}\left(\mathrm{SNR}\right)\right)$ in R(SNR), defined as the limiting ratios of $R_{1}\left(\mathrm{SNR}\right)$ and $R_{2}\left(\mathrm{SNR}\right)$ to the logarithm of the SNR as the SNR tends to infinity. More precisely, the pre-log region is defined as the set of all pre-log pairs $\left(\Pi_{R_{1}},\Pi_{R_{2}}\right)$ for which there exists a sequence of rate pairs $\left(R_{1}\left(\mathrm{SNR}\right),R_{2}\left(\mathrm{SNR}\right)\right)$ that, for every SNR, lies in R(SNR) and satisfies

(47) $$\begin{array}{cc} \underset{\mathrm{SNR}\to \infty }{lim sup}\frac{R_{1}\left(\mathrm{SNR}\right)}{\log\mathrm{SNR}} & =\Pi_{R_{1}} \end{array}$$

(48) $$\begin{array}{cc} \underset{\mathrm{SNR}\to \infty }{lim sup}\frac{R_{2}\left(\mathrm{SNR}\right)}{\log\mathrm{SNR}} & =\Pi_{R_{2}}. \end{array}$$

Let the maximum achievable pre-logs be defined as

(49) $$\begin{array}{cc} \Pi_{R_{1}^{*}} & ≜\underset{\mathrm{SNR}\to \infty }{lim sup}\;\frac{R_{1}^{*}\left(\mathrm{SNR}\right)}{\log\mathrm{SNR}} \end{array}$$

(50) $$\begin{array}{cc} \Pi_{R_{2}^{*}} & ≜\underset{\mathrm{SNR}\to \infty }{lim sup}\;\frac{R_{2}^{*}\left(\mathrm{SNR}\right)}{\log\mathrm{SNR}} \end{array}$$

(51) $$\begin{array}{cc} \Pi_{R_{1+2}^{*}} & ≜\underset{\mathrm{SNR}\to \infty }{lim sup}\;\frac{R_{1+2}^{*}\left(\mathrm{SNR}\right)}{\log\mathrm{SNR}} \end{array}$$

and define the capacity pre-logs $\Pi_{C_{1}}$, $\Pi_{C_{2}}$, and $\Pi_{C_{1+2}}$ in the same way but with $R_{1}^{*}\left(\mathrm{SNR}\right)$, $R_{2}^{*}\left(\mathrm{SNR}\right)$, and $R_{1+2}^{*}\left(\mathrm{SNR}\right)$ replaced by the respective capacities $C_{1}\left(\mathrm{SNR}\right)$, $C_{2}\left(\mathrm{SNR}\right)$, and $C_{1+2}\left(\mathrm{SNR}\right)$. If the ratios of the rates to logSNR in (47)–(51) converge as SNR→∞, i.e., if the limits superior are, in fact, limits, then the pre-log region is given by the set

(52) $$\begin{array}{c} \Pi_{R}=\left\{\left(\Pi_{R_{1}},\Pi_{R_{2}}\right):\Pi_{R_{1}}\leq \Pi_{R_{1}^{*}},\;\Pi_{R_{2}}\leq \Pi_{R_{2}^{*}},\;\Pi_{R_{1}}+\Pi_{R_{2}}\leq \Pi_{R_{1+2}^{*}}\right\}. \end{array}$$

Indeed, R(SNR) includes all rate pairs $\left(R_{1}\left(\mathrm{SNR}\right),R_{2}\left(\mathrm{SNR}\right)\right)$ satisfying

$$\begin{array}{ccc} (53) & \frac{R_{1}\left(\mathrm{SNR}\right)}{\log\mathrm{SNR}} & \leq \frac{R_{1}^{*}\left(\mathrm{SNR}\right)}{\log\mathrm{SNR}}\\ (54) & \frac{R_{2}\left(\mathrm{SNR}\right)}{\log\mathrm{SNR}} & \leq \frac{R_{2}^{*}\left(\mathrm{SNR}\right)}{\log\mathrm{SNR}}\\ (55) & \frac{R_{1}\left(\mathrm{SNR}\right)}{\log\mathrm{SNR}}+\frac{R_{2}\left(\mathrm{SNR}\right)}{\log\mathrm{SNR}} & \leq \frac{R_{1+2}^{*}\left(\mathrm{SNR}\right)}{\log\mathrm{SNR}}. \end{array}$$

This implies that, for every pre-log pair $\left(\Pi_{R_{1}},\Pi_{R_{2}}\right)$ in $\Pi_{R}$, we can find a sequence of rate pairs $\left(R_{1}\left(\mathrm{SNR}\right),R_{2}\left(\mathrm{SNR}\right)\right)$ in R(SNR) that achieve (47)–(48). Conversely, if the pre-log pair $\left(\Pi_{R_{1}},\Pi_{R_{2}}\right)$ does not lie in $\Pi_{R}$, then there exists a sufficiently large ${\mathrm{SNR}}_{0}$ such that, for all $\mathrm{SNR}\geq {\mathrm{SNR}}_{0}$, at least one of the three conditions (53)–(55) is violated. Consequently, we cannot find a sequence of rate pairs $\left(R_{1}\left(\mathrm{SNR}\right),R_{2}\left(\mathrm{SNR}\right)\right)$ in R(SNR) that satisfies (47)–(48).

We next present our result on the pre-log region of the two-user MIMO fading MAC achievable with the joint-transmission scheme.

**Theorem** **2.** *Consider the MIMO fading MAC *(40)*. Then, the joint-transmission scheme achieves the pre-log region*

(56) $$\begin{array}{ccc} \{ & (\Pi_{R_{1}},\Pi_{R_{2}}): & \;\;\Pi_{R_{1}}\leq \min\left(n_{r},n_{t,1}\right)\left(1-\frac{n_{t,1}+n_{t,2}}{L^{*}}\right),\\ \; & & \;\;\Pi_{R_{2}}\leq \min\left(n_{r},n_{t,2}\right)\left(1-\frac{n_{t,1}+n_{t,2}}{L^{*}}\right),\\ \; & & \;\;\Pi_{R_{1}}+\Pi_{R_{2}}\leq \min\left(n_{r},n_{t,1}+n_{t,2}\right)\left(1-\frac{n_{t,1}+n_{t,2}}{L^{*}}\right)\} \end{array}$$

where $L^{*}=\left\lfloor \frac{1}{2\lambda_{D}}\right\rfloor$.

**Proof.**  See Section 6. □

The pre-log region given in Theorem 2 is the largest region achievable with any transmission scheme that uses $\left(n_{t,1}+n_{t,2}\right)/L^{*}$ of the time instants for transmitting pilot symbols. Indeed, even if the channel estimator would be able to estimate the fading coefficients perfectly, and even if we could decode the data symbols using a maximum-likelihood decoder, the capacity pre-log region (without pilot transmission) would be given by the set [1,2,24]

(57) $$\begin{array}{c} \left\{(\Pi_{R_{1}},\Pi_{R_{2}}):\Pi_{R_{1}}\leq \min\left(n_{r},n_{t,1}\right),\;\Pi_{R_{2}}\leq \min\left(n_{r},n_{t,2}\right),\;\Pi_{R_{1}}+\Pi_{R_{2}}\leq \min\left(n_{r},n_{t,1}+n_{t,2}\right)\right\} \end{array}$$

which, after multiplying by $1-\left(n_{t,1}+n_{t,2}\right)/L^{*}$ to account for the transmission of pilot symbols, becomes (56). Thus, in order to improve upon (56), one would need to design a transmission scheme that employs less than $\left(n_{t,1}+n_{t,2}\right)/L^{*}$ pilot symbols per channel use.

**Remark** **2** (TDMA Pre-Log). *Consider the MIMO fading MAC *(40)*. Then, the TDMA scheme employing nearest neighbor decoding and pilot-aided channel estimation achieves the pre-log region*

(58) $$\begin{array}{ccc} \{ & (\Pi_{R_{1}},\Pi_{R_{2}}): & \;\;\Pi_{R_{1}}\leq \beta \min\left(n_{r},n_{t,1}\right)\left(1-\frac{n_{t,1}}{L^{*}}\right),\\ \; & & \;\;\Pi_{R_{2}}\leq \left(1-\beta \right)\min\left(n_{r},n_{t,2}\right)\left(1-\frac{n_{t,2}}{L^{*}}\right),\;0\leq \beta \leq 1\} \end{array}$$

where $L^{*}=\left\lfloor \frac{1}{2\lambda_{D}}\right\rfloor$. This follows directly from the pre-log of the point-to-point MIMO fading channel (Theorem 1) with the number of transmit antennas given by $n_{t,1}$ and $n_{t,2}$, respectively.

Please note that the sum of the pre-logs $\Pi_{R_{1}}+\Pi_{R_{2}}$ is upper-bounded by the capacity pre-log of the point-to-point MIMO fading channel with $\left(n_{t,1}+n_{t,2}\right)$ transmit antennas and $n_{r}$ receive antennas, since the latter channel corresponds to the case where the transmitting terminals can cooperate. While the capacity pre-log of general point-to-point MIMO fading channels remains an open problem, the capacity pre-log of point-to-point MISO fading channels is known, cf. (22). It thus follows that, for $n_{r}=n_{t,1}=n_{t,2}=1$, we have

(59) $$\Pi_{R_{1}}+\Pi_{R_{2}}\leq \Pi_{C_{1+2}}=1-2\lambda_{D}$$

which together with the single-user constraints

(60) $$\begin{array}{cc} \Pi_{R_{1}} & \leq \Pi_{C_{1}}=1-2\lambda_{D} \end{array}$$

(61) $$\begin{array}{cc} \Pi_{R_{2}} & \leq \Pi_{C_{2}}=1-2\lambda_{D} \end{array}$$

implies that TDMA achieves the capacity pre-log region of the SISO fading MAC. The next section provides a more detailed comparison between the joint-transmission scheme and TDMA.

### 4.2. Joint Transmission Versus TDMA

In this section, we discuss how the joint-transmission scheme performs compared to TDMA. To this end, we compare the sum-rate pre-log $\Pi_{R_{1+2}^{*}}$ of the joint-transmission scheme (Theorem 2) with the sum-rate pre-log of the TDMA scheme employing nearest neighbor decoding and pilot-aided channel estimation (Remark 2) as well as with the sum-rate pre-log of the coherent TDMA scheme, where the receiver has knowledge of the realizations of the fading processes $\left\{H_{s,k},\;k\in \mathbb{Z}\right\}$, s=1,2. In the latter case, the sum-rate pre-log is given by

(62) $$\Pi_{R_{1+2}^{*}}=\beta \min\left(n_{r},n_{t,1}\right)+\left(1-\beta \right)\min\left(n_{r},n_{t,2}\right).$$

The following corollary presents a sufficient condition on $L^{*}$ under which the sum-rate pre-log of the joint-transmission scheme is strictly larger than that of the coherent TDMA scheme (62), as well as a sufficient condition on $L^{*}$ under which the sum-rate pre-log of the joint-transmission scheme is strictly smaller than the sum-rate pre-log of the TDMA scheme given in Remark 2. Since (62) is an upper bound on the sum-rate pre-log of any TDMA scheme over the MIMO fading MAC (40), and since the sum-rate pre-log given in Remark 2 is a lower bound on the sum-rate pre-log of the best TDMA scheme, it follows that the sufficient conditions presented in Corollary 1 hold also for the best TDMA scheme.

**Corollary** **1.** *Consider the MIMO fading MAC *(40)*. The joint-transmission scheme achieves a larger sum-rate pre-log than any TDMA scheme if*

(63) $$L^{*}>\frac{\min\left(n_{r},n_{t,1}+n_{t,2}\right)\left(n_{t,1}+n_{t,2}\right)}{\min\left(n_{r},n_{t,1}+n_{t,2}\right)-\min\left(n_{r},\max\left(n_{t,1},n_{t,2}\right)\right)}$$

*where we define a/0≜∞ for every a>0. Conversely, the best TDMA scheme achieves a larger sum-rate pre-log than the joint-transmission scheme if*

(64) $$\begin{array}{cc} L^{*}<\; & \frac{\min\left(n_{r},n_{t,1}+n_{t,2}\right)\left(n_{t,1}+n_{t,2}\right)}{\min\left(n_{r},n_{t,1}+n_{t,2}\right)-\min\left(n_{r},n_{t,1},n_{t,2}\right)}-\frac{\min(n_{t,1}n_{r},{n_{t,1}}^{2},n_{t,2}n_{r},{n_{t,2}}^{2})}{\min\left(n_{r},n_{t,1}+n_{t,2}\right)-\min\left(n_{r},n_{t,1},n_{t,2}\right)}. \end{array}$$

Recall that $L^{*}$ is inversely proportional to the bandwidth of the power spectral density $f_{H}\left(\cdot \right)$, which in turn is inversely proportional to the coherence time of the fading channel. Corollary 1 thus demonstrates that the joint-transmission scheme tends to be superior to TDMA when the coherence time of the channel is large. In contrast, TDMA is superior to the joint-transmission scheme when the coherence time of the channel is small. Intuitively, this can be explained by observing that, compared to TDMA, the joint-transmission scheme uses the antennas at the transmitters and at the receiver more efficiently, but requires more pilot symbols to estimate the fading coefficients. Thus, when the coherence time is large, the number of pilot symbols required to estimate the fading is small, so the gain in achievable rate by using the antennas more efficiently dominates the loss incurred by requiring more pilot symbols. On the other hand, when the coherence time is small, the number of pilot symbols required to estimate the fading coefficients is large and the loss in achievable rate incurred by requiring more pilot symbols dominates the gain by using the antennas more efficiently.

We next evaluate (63) and (64) for some particular values of $n_{r}$, $n_{t,1}$, and $n_{t,2}$.

#### 4.2.1. Receiver Employs Less Antennas than Transmitters

Suppose that $n_{r}\leq \min\left(n_{t,1},n_{t,2}\right)$. Then, the right-hand sides (RHSs) of (63) and (64) become *∞*, so every finite $L^{*}$ satisfies (64). Thus, if the number of receive antennas is smaller than the number of transmit antennas, then, irrespective of $L^{*}$, TDMA is superior to the joint-transmission scheme.

#### 4.2.2. Receiver Employs More Antennas than Transmitters

Suppose that $n_{r}\geq n_{t,1}+n_{t,2}$, and suppose that $n_{t,1}=n_{t,2}=n_{t}$. Then, (63) and (64) become

(65) $$L^{*}>4n_{t}$$

and

(66) $$L^{*}<3n_{t}.$$

**Figure 5:**
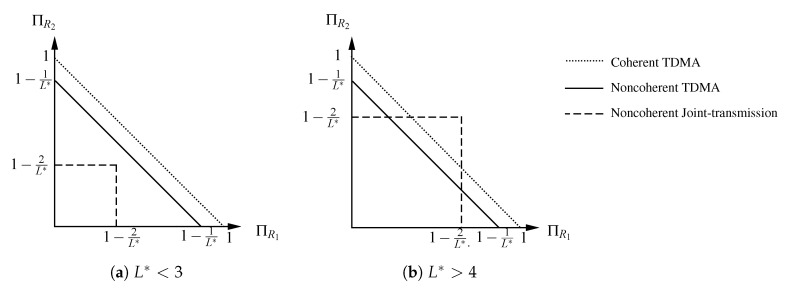
Pre-log regions for a fading MAC with $n_{r}=2$ and $n_{t,1}=n_{t,2}=1$ for different values of $L^{*}$. Depicted are the pre-log region for the joint-transmission scheme as given in Theorem 2 (dashed line), the pre-log region of the TDMA scheme as given in Remark 2 (solid line), and the pre-log region of the coherent TDMA scheme (62) (dotted line).

Thus, if $L^{*}$ is greater than $4n_{t}$, then the joint-transmission scheme is superior to TDMA. In contrast, if $L^{*}$ is smaller than $3n_{t}$, then TDMA is superior. This is illustrated in Figure 5 for the case where $n_{r}=2$ and $n_{t,1}=n_{t,2}=1$. Please note that if $L^{*}$ is between $3n_{t}$ and $4n_{t}$, then the joint-transmission scheme is superior to the TDMA scheme presented in Remark 2, but it may be inferior to the best TDMA scheme.

#### 4.2.3. A Case in between

Suppose that $n_{r}\leq n_{t,1}+n_{t,2}$ and $n_{t,2}<n_{r}\leq n_{t,1}$. Then, (63) and (64) become

(67) $$L^{*}>\infty$$

and

(68) $$L^{*}<n_{t,2}+\frac{n_{r}n_{t,1}}{n_{r}-n_{t,2}}.$$

Thus, in this case the joint-transmission scheme is always inferior to the coherent TDMA scheme (62), but it can be superior to the TDMA scheme in Remark 2.

### 4.3. Typical Values of $L^{*}$

We briefly discuss the range of values of $L^{*}$ that may occur in practical scenarios. To this end, we first recall that $L^{*}=\left\lfloor 1/\left(2\lambda_{D}\right)\right\rfloor$, and that $\lambda_{D}$ is the bandwidth of the fading power spectral density $f_{H}\left(\cdot \right)$, which can be associated with the Doppler spread of the channel as [12]

(69) $$\lambda_{D}=\frac{f_{m}}{W_{c}}.$$

Here $f_{m}$ is the maximum Doppler shift given by

(70) $$f_{m}=\frac{v}{c}f_{c}$$

where *v* is the speed of the mobile device, $c=3\cdot 10^{8}$ m/s is the speed of light, and $f_{c}$ is the carrier frequency; and $W_{c}$ is the coherence bandwidth of the channel approximated as [12,25]

(71) $$W_{c}\approx \frac{1}{5\sigma_{\tau }}$$

where $\sigma_{\tau }$ is the delay spread. Following the order-of-magnitude computations of Etkin and Tse [12], we determine typical values of $\lambda_{D}$ for indoor, urban, and hilly area environments and for carrier frequencies ranging from 800 MHz to 5 GHz and tabulate the results in Table 1.

For indoor environments and mobile speeds of 5 km/h, we have that $L^{*}$ is typically larger than $5\times 10^{4}$. For urban environments, $L^{*}$ is typically larger than $2.5\times 10^{3}$ for mobile speeds of 5 km/h and larger than 125 for mobile speeds of 75 km/h. For hilly area environments and mobile speeds of 200 km/h, $L^{*}$ ranges typically from 10 to 250. Thus, for most practical scenarios, $L^{*}$ is typically large. It therefore follows that, if $n_{r}\geq n_{t,1}+n_{t,2}$, the condition (63) is satisfied unless $n_{t,1}+n_{t,2}$ is very large. For example, if the receiver employs more antennas than the transmitters, and if $n_{t,1}=n_{t,2}=n_{t}$, then $L^{*}>4n_{t}$ is satisfied even for urban environments and mobile speeds of 75 km/h, as long as $n_{t}<30$. Only for hilly area environments and mobile speeds of 200 km/h, this condition may not be satisfied for a practical number of transmit antennas. Thus, if the number of antennas at the receiver is sufficiently large, then the joint-transmission scheme is superior to TDMA in most practical scenarios. On the other hand, if $n_{r}\leq \min\left(n_{t,1},n_{t,2}\right)$, then TDMA is always superior to the joint-transmission scheme, irrespective of how large $L^{*}$ is. This suggests that one should use more antennas at the receiver than at the transmitters.

## 5. Proof of Theorem 1

Theorem 1 is proved as follows. We first characterize the estimation error from the linear interpolator (7). We then compute the rates achievable with the communication scheme described in Section 2. Finally, we analyze the pre-log corresponding to these rates.

### 5.1. Linear Interpolator

We first note that the estimate of $H_{k}\left(r,t\right)$ is given by (7), namely,

(72) $${\hat{H}}_{k}^{\left(T\right)}\left(r,t\right)=\sum_{\begin{array}{c} k^{\prime }=k-TL:\\ k^{\prime }\in P \end{array}}^{k+TL}a_{k^{\prime }}\left(r,t\right)Y_{k^{\prime }}\left(r\right),\;k\in D.$$

We denote the interpolation error by $E_{k}^{\left(T\right)}\left(r,t\right)=H_{k}\left(r,t\right)-{\hat{H}}_{k}^{\left(T\right)}\left(r,t\right)$.

For future reference, and for any k∈, we express k=jL+ℓ, so ℓ=kmodL. Assuming that the first pilot symbol is transmitted at k=0, it follows that $\ell =0,\cdots ,n_{t}-1$ for k∈P and $\ell =n_{t},\cdots ,L-1$ for k∈D. The statistical properties of the channel estimator for a given window size *T* are summarized in the following lemma.

**Lemma** **1.** *For a given T, the linear interpolator *(72)* has the following properties.*

*1.*  *For each $t=1,\cdots ,n_{t}$, $r=1,\cdots ,n_{r}$, and $\ell =n_{t},\cdots ,L-1$, the estimate ${\hat{H}}_{jL+\ell }^{\left(T\right)}\left(r,t\right)$ and the corresponding estimation error $E_{jL+\ell }^{\left(T\right)}\left(r,t\right)$ are independent zero-mean complex-Gaussian random variables.*
*2.*  *(a)  For a given transmit antenna t and $\ell \in \{n_{t},\cdots ,L-1\}$, the $n_{r}$ processes*
  (73) $$\left\{\left({\hat{H}}_{jL+\ell }^{\left(T\right)}\left(1,t\right),E_{jL+\ell }^{\left(T\right)}\left(1,t\right)\right),\;j\in \mathbb{Z}\right\},\cdots ,\left\{\left({\hat{H}}_{jL+\ell }^{\left(T\right)}\left(n_{r},t\right),E_{jL+\ell }^{\left(T\right)}\left(n_{r},t\right)\right),\;j\in \mathbb{Z}\right\}$$
  *are independent and have the same law.*
  *(b)*  *For a given receive antenna r and $\ell \in \{n_{t},\cdots ,L-1\}$, the $n_{t}$ processes*
    (74) $$\left\{\left({\hat{H}}_{jL+\ell }^{\left(T\right)}\left(r,1\right),E_{jL+\ell }^{\left(T\right)}\left(r,1\right)\right),\;j\in \mathbb{Z}\right\},\cdots ,\left\{\left({\hat{H}}_{jL+\ell }^{\left(T\right)}\left(r,n_{t}\right),E_{jL+\ell }^{\left(T\right)}\left(r,n_{t}\right)\right),\;j\in \mathbb{Z}\right\}$$
    *are independent but have different laws.*

*3.*  *For each $\ell =n_{t},\cdots ,L-1$, the process $\left\{\left({\hat{H}}_{jL+\ell }^{\left(T\right)},\;H_{jL+\ell },\;Z_{jL+\ell },\;X_{jL+\ell }\right),\;j\in \mathbb{Z}\right\}$ is jointly stationary and ergodic.*
*4.*  *For $\ell =n_{t},\cdots ,L-1$, it holds that*
  (75) $$E\left[Z_{\ell }^{†}{\hat{H}}_{\ell }^{\left(T\right)}X_{\ell }\right]=0$$
  *where ${\left(\cdot \right)}^{†}$ denotes the conjugate transpose.*

**Proof.**  See Appendix A. □

### 5.2. Achievable Rates and Pre-Logs

In the following proof, we only consider the case where $n_{t}=n_{r}$. The more general case of $n_{t}\neq n_{r}$ follows then by using only $n_{r}$ transmit antennas or by ignoring $n_{r}-n_{t}$ antennas at the receiver. This yields a lower bound on the maximum achievable rate and does not incur a loss with respect to the pre-log. Indeed, it can be shown that the nearest neighbor decoder described in Section 2 achieves the pre-log $\min(n_{r},n_{t})$. Thus, increasing $n_{t}$ beyond $n_{r}$ or $n_{r}$ beyond $n_{t}$ does not improve the pre-log achievable by such a decoder. In fact, increasing $n_{t}$ beyond $n_{r}$ requires the transmission of more pilot symbols and does therefore even reduce the pre-log achievable with the communication scheme described in Section 2.

To prove Theorem 1, we analyze the generalized mutual information (GMI) [27] for the channel and communication scheme in Section 2. The GMI, denoted by $I_{T}^{\mathrm{gmi}}\left(\mathrm{SNR}\right)$, specifies the highest information rate for which the average probability of error, averaged over the ensemble of i.i.d. Gaussian codebooks, tends to zero as the codeword length *n* tends to infinity (see [7,16,17] and references therein). The GMI for stationary Gaussian fading channels employing nearest neighbor decoding has been evaluated in [16,17] for the case where a genie provides the receiver with an estimate of the fading process. However, the estimate considered in [16,17] is assumed to be jointly stationary and ergodic with $\left\{\left(H_{k},X_{k},Z_{k}\right),\;k\in \mathbb{Z}\right\}$, which is not satisfied by $\left\{{\hat{H}}_{k}^{\left(T\right)},\;k\in D\right\}$. We therefore need to adapt the work in [16,17] to our channel model. For completeness, we present all the main steps here, even though they are very similar to the ones in [16,17].

We prove Theorem 1 as follows:

1. We compute a lower bound on $I_{T}^{\mathrm{gmi}}\left(\mathrm{SNR}\right)$ for a fixed window size *T* (Section 5.2.1).
2. We analyze the behavior of this lower bound as *T* tends to infinity (Section 5.2.2).
3. We evaluate the limiting ratio of this lower bound to logSNR as SNR tends to infinity (Section 5.2.3).

#### 5.2.1. $I_{T}^{\mathrm{gmi}}\left(\mathrm{SNR}\right)$ for a Fixed *T*

We analyze the GMI for a fixed *T* using a random coding upper bound on the average error probability. Please note that due to the symmetry of the codebook construction, it suffices to consider the error behavior conditioned on the event that message 1 was transmitted. Let $E(m^{\prime })$ denote the event that $D\left(m^{\prime }\right)\leq D\left(1\right)$, where D(·) was defined in (17). The ensemble-average error probability—where the average is over the ensemble of i.i.d. Gaussian codes—corresponding to message m=1 is thus given by

(76) $${\bar{P}}_{e}\left(1\right)=\mathrm{Pr}\left\{\underset{m^{\prime }\neq 1}{⋃}E\left(m^{\prime }\right)\right\}.$$

To evaluate the GMI from the RHS of (76), we next define some useful quantities. Recall the channel and transmission model in Section 2. Without loss of generality, assume that the first pilot vector is transmitted at time k=0. Let

(77) $$\begin{array}{cc} F(\mathrm{SNR}) & ≜n_{r}+\frac{\mathrm{SNR}}{\left(L-n_{t}\right)n_{t}}\sum_{\ell =n_{t}}^{\left(L-1\right)}E\left[{∥E_{\ell }^{\left(T\right)}∥}_{F}^{2}\right] \end{array}$$

where $E_{\ell }^{\left(T\right)}$ is a random matrix whose row-*r* column-*t* entry is given by $E_{\ell }^{\left(T\right)}\left(r,t\right)$, and where ${∥\cdot ∥}_{F}$ denotes the Frobenius norm. For some arbitrary δ>0, we further define the typical set

(78) $$\begin{array}{c} T_{\delta }≜\{\left(x_{k},y_{k},{\hat{H}}_{k}^{\left(T\right)}\right),k=0,\cdots ,n^{\prime }-1:\left|\frac{1}{n}\sum_{k\in D^{\left(n^{\prime }\right)}}{∥y_{k}-\sqrt{\frac{\mathrm{SNR}}{n_{t}}}\;{\hat{H}}_{k}^{\left(T\right)}x_{k}∥}^{2}-F\left(\mathrm{SNR}\right)\right|<\delta \} \end{array}$$

with $D^{\left(n^{\prime }\right)}=\left\{0,\cdots ,n^{\prime }-1\right\}\cap D$ and $n^{\prime }=n_{p}+n+n_{g}$, as given in (18) and (4), respectively. Then, we have the following convergence as *n* tends to infinity.

**Lemma** **2.** *For the channel model and communication scheme described in Section 2, we have that*

(79) $$\lim_{n\to \infty }\mathrm{Pr}\left\{\left(X^{n^{\prime }},Y^{n^{\prime }},{\hat{H}}^{\left(T\right),n^{\prime }}\right)\in T_{\delta }\right\}=1,\;\forall \delta >0$$

*where we have used the notation $U^{n^{\prime }}$ to denote the sequence $U_{0},\cdots ,U_{n^{\prime }-1}$.*

**Proof.**  We have

$$\begin{array}{ccc} & \lim_{n\to \infty } & \frac{1}{n}\sum_{k\in D^{\left(n^{\prime }\right)}}{∥y_{k}-\sqrt{\frac{\mathrm{SNR}}{n_{t}}}\;{\hat{H}}_{k}^{\left(T\right)}x_{k}∥}^{2}\\ (80)\; & & =\lim_{n\to \infty }\frac{1}{n}\sum_{k\in D^{\left(n^{\prime }\right)}}{∥\sqrt{\frac{\mathrm{SNR}}{n_{t}}}\;\left(H_{k}-{\hat{H}}_{k}^{\left(T\right)}\right)x_{k}+z_{k}∥}^{2}\\ (81)\; & & =\frac{1}{L-n_{t}}\sum_{\ell =n_{t}}^{L-1}\lim_{n\to \infty }\frac{L-n_{t}}{n}\sum_{j=0}^{\frac{n}{L-n_{t}}-1}{∥\sqrt{\frac{\mathrm{SNR}}{n_{t}}}\left(H_{jL+\ell }-{\hat{H}}_{jL+\ell }^{\left(T\right)}\right)x_{jL+\ell }+z_{jL+\ell }∥}^{2}\\ (82)\; & & =\frac{1}{L-n_{t}}\sum_{\ell =n_{t}}^{L-1}E\left[{∥\sqrt{\frac{\mathrm{SNR}}{n_{t}}}\left(H_{\ell }-{\hat{H}}_{\ell }^{\left(T\right)}\right){\bar{X}}_{\ell }+Z_{\ell }∥}^{2}\right],\;\mathrm{almost}\;\mathrm{surely}\\ (83)\; & & =\frac{1}{L-n_{t}}\sum_{\ell =n_{t}}^{L-1}\left(n_{r}+\frac{\mathrm{SNR}}{n_{t}}E\left[{∥E_{\ell }^{\left(T\right)}{\bar{X}}_{\ell }∥}^{2}\right]\right)\\ (84)\; & & =F(\mathrm{SNR}). \end{array}$$

Herein (82) follows from (Part 3) of Lemma 1 and the ergodic theorem ([28], Chapter 7); (83) follows from (Part 4) of Lemma 1; and (84) follows since ${\bar{X}}_{\ell }$ has zero mean and covariance matrix $I_{n_{t}}$, and is independent from $E_{\ell }^{\left(T\right)}$ (since $\left\{E_{k}^{\left(T\right)},\;k\in D\right\}$ is a function of $\left\{\left(H_{k},Z_{k}\right),k\in \mathbb{Z}\right\}$). It thus follows that, as n→∞,

(85) $$\frac{1}{n}\sum_{k\in D^{\left(n^{\prime }\right)}}{∥y_{k}-\sqrt{\frac{\mathrm{SNR}}{n_{t}}}\;{\hat{H}}_{k}^{\left(T\right)}x_{k}∥}^{2}$$

converges to F(SNR) almost surely, which in turn implies that it also converges in probability, which is (79). □

Considering the typical set (78), and following the derivation in [16,17], the error probability ${\bar{P}}_{e}\left(1\right)$ in (76) can be upper-bounded as

(86) $$\begin{array}{cc} {\bar{P}}_{e}\left(1\right)\leq & \;\;e^{nR}\cdot \mathrm{Pr}\left\{\left.\frac{1}{n}\cdot D\left(m^{\prime }\right)<F\left(\mathrm{SNR}\right)+\delta \right|\left(X^{n^{\prime }}\left(1\right),Y^{n^{\prime }},{\hat{H}}^{\left(T\right),n^{\prime }}\right)\in T_{\delta }\right\}\\ \; & +\mathrm{Pr}\left\{\left(X^{n^{\prime }}\left(1\right),Y^{n^{\prime }},{\hat{H}}^{\left(T\right),n^{\prime }}\right)\in T_{\delta }^{c}\right\},\;m^{\prime }\neq 1 \end{array}$$

where $T_{\delta }^{c}$ denotes the complement of $T_{\delta }$. It follows from Lemma 2 that the second term on the RHS of (86) can be made arbitrarily small by letting *n* tend to infinity.

The GMI characterizes the rate of exponential decay of the expression

(87) $$\mathrm{Pr}\left\{\left.\frac{1}{n}\cdot D\left(m^{\prime }\right)<F\left(\mathrm{SNR}\right)+\delta \right|\left(X^{n^{\prime }}\left(1\right),Y^{n^{\prime }},{\hat{H}}^{\left(T\right),n^{\prime }}\right)\in T_{\delta }\right\},\;m^{\prime }\neq 1$$

as n→∞ [16,17]. The computation of the GMI requires the conditional log moment-generating function of the metric $D(m^{\prime })$ associated with the wrong message output $m^{\prime }\neq 1$, conditioned on the channel outputs and on the fading estimates, which is defined as

(88) $$\begin{array}{cc} \kappa_{n}\left(\theta ,y^{n^{\prime }},{\hat{H}}^{\left(T\right),n^{\prime }}\right) & ≜\log E\left[\left.\exp\left(\frac{\theta }{n}\sum_{k\in D^{\left(n^{\prime }\right)}}D_{k}\left(m^{\prime }\right)\right)\right|\left\{\left(y_{k},{\hat{H}}_{k}^{\left(T\right)}\right),\;k\in D^{\left(n^{\prime }\right)}\right\}\right] \end{array}$$

where

(89) $$D_{k}\left(m^{\prime }\right)≜{∥y_{k}-\sqrt{\frac{\mathrm{SNR}}{n_{t}}}\;{\hat{H}}_{k}^{\left(T\right)}x_{k}\left(m^{\prime }\right)∥}^{2}.$$

Proceeding along the lines of [16,17], we can express the conditional log moment-generating function in (88) as the sum of conditional log moment-generating functions for the individual vector metrics $D_{k}\left(m^{\prime }\right)$, $k\in D^{\left(n^{\prime }\right)}$, i.e.,

$$\begin{array}{cc} \; & \kappa_{n}\left(\theta ,y^{n^{\prime }},{\hat{H}}^{\left(T\right),n^{\prime }}\right)\\ (90)\; & \;=\sum_{k\in D^{\left(n^{\prime }\right)}}\log E\left[\left.\exp\left(\frac{\theta }{n}D_{k}\left(m^{\prime }\right)\right)\right|y_{k},{\hat{H}}_{k}^{\left(T\right)}\right]\\ (91)\; & \;=\sum_{k\in D^{\left(n^{\prime }\right)}}\left(\frac{\theta }{n}y_{k}^{†}{\left(I_{n_{r}}-\frac{\theta }{n}\frac{\mathrm{SNR}}{n_{t}}{\hat{H}}_{k}^{\left(T\right)}{\hat{H}}_{k}^{†(T)}\right)}^{-1}y_{k}-\log\det\left(I_{n_{r}}-\frac{\theta }{n}\frac{\mathrm{SNR}}{n_{t}}{\hat{H}}_{k}^{\left(T\right)}{\hat{H}}_{k}^{†(T)}\right)\right). \end{array}$$

We then have that, for all θ<0,

$$\begin{array}{cc} \; & \lim_{n\to \infty }\;\frac{1}{n}\cdot \kappa_{n}\left(n\theta ,y^{n^{\prime }},{\hat{H}}^{\left(T\right),n^{\prime }}\right)\\ \; & \;=\lim_{n\to \infty }\frac{1}{n}\sum_{k\in D^{\left(n^{\prime }\right)}}\theta y_{k}^{†}{\left(I_{n_{r}}-\theta \frac{\mathrm{SNR}}{n_{t}}{\hat{H}}_{k}^{\left(T\right)}{\hat{H}}_{k}^{†(T)}\right)}^{-1}y_{k}\\ (92)\; & \;\;\;-\lim_{n\to \infty }\frac{1}{n}\sum_{k\in D^{\left(n^{\prime }\right)}}\log\det\left(I_{n_{r}}-\theta \frac{\mathrm{SNR}}{n_{t}}{\hat{H}}_{k}^{\left(T\right)}{\hat{H}}_{k}^{†(T)}\right)\\ \; & \;=\frac{1}{L-n_{t}}\sum_{\ell =n_{t}}^{L-1}\lim_{n\to \infty }\frac{L-n_{t}}{n}\sum_{j=0}^{\frac{n}{L-n_{t}}-1}\theta y_{jL+\ell }^{†}{\left(I_{n_{r}}-\theta \frac{\mathrm{SNR}}{n_{t}}{\hat{H}}_{jL+\ell }^{\left(T\right)}{\hat{H}}_{jL+\ell }^{†(T)}\right)}^{-1}y_{jL+\ell }\\ (93)\; & \;\;-\frac{1}{L-n_{t}}\sum_{\ell =n_{t}}^{L-1}\lim_{n\to \infty }\;\frac{L-n_{t}}{n}\sum_{j=0}^{\frac{n}{L-n_{t}}-1}\log\det\left(I_{n_{r}}-\theta \frac{\mathrm{SNR}}{n_{t}}{\hat{H}}_{jL+\ell }^{\left(T\right)}{\hat{H}}_{jL+\ell }^{†(T)}\right)\\ \; & \;=\frac{1}{L-n_{t}}\sum_{\ell =n_{t}}^{L-1}E\left[\theta Y_{\ell }^{†}\cdot {\left(I_{n_{r}}-\theta \frac{\mathrm{SNR}}{n_{t}}{\hat{H}}_{\ell }^{\left(T\right)}{\hat{H}}_{\ell }^{†(T)}\right)}^{-1}\cdot Y_{\ell }\right]\\ (94)\; & \;\;\;-\;\frac{1}{L-n_{t}}\sum_{\ell =n_{t}}^{L-1}E\left[\log\det\left(I_{n_{r}}-\theta \frac{\mathrm{SNR}}{n_{t}}{\hat{H}}_{\ell }^{\left(T\right)}{\hat{H}}_{\ell }^{†(T)}\right)\right],\;\mathrm{almost}\;\mathrm{surely}\\ (95)\; & \;≜\kappa (\theta ,\mathrm{SNR}) \end{array}$$

where the last step should be regarded as the definition of κ(θ,SNR). The convergence in (94) is due to the ergodicity of $\left\{\left(Y_{jL+\ell },\;{\hat{H}}_{jL+\ell }^{\left(T\right)}\right),\;j\in \mathbb{Z}\right\}$, $\ell =n_{t},\cdots ,L-1$ (see (Part 3) of Lemma 1) and the ergodic theorem.

Following the same steps as in [16,17], we can then show that for all $\delta^{\prime }>0$, the ensemble-average error probability can be bounded as

(96) $${\bar{P}}_{e}\left(1\right)\leq \exp\left(nR\right)\exp\left(-n\left(I_{T}^{\mathrm{gmi}}\left(\mathrm{SNR}\right)-\delta^{\prime }\right)\right)+\varepsilon \left(\delta^{\prime },n\right)$$

for some $\varepsilon (\delta^{\prime },n)$ satisfying

(97) $$\lim_{n\to \infty }\varepsilon \left(\delta^{\prime },n\right)=0,\;\delta^{\prime }>0.$$

On the RHS of (96), $I_{T}^{\mathrm{gmi}}\left(\mathrm{SNR}\right)$ denotes the GMI as a function of the SNR for a fixed *T*, which is given by

(98) $$I_{T}^{\mathrm{gmi}}\left(\mathrm{SNR}\right)=\frac{L-n_{t}}{L}\left(\underset{\theta <0}{\sup}\;\left(\theta F(\mathrm{SNR})-\kappa (\theta ,\mathrm{SNR})\right)\right).$$

Herein the pre-factor $(L-n_{t})/L$ equals the fraction of time instants used for data transmission. The bound (96) implies that for rates below $I_{T}^{\mathrm{gmi}}\left(\mathrm{SNR}\right)$, the communication scheme described in Section 2 has vanishing error probability as *n* tends to infinity.

Combining (77) and (94) with (98) yields

(99) $$\begin{array}{cc} I_{T}^{\mathrm{gmi}}\left(\mathrm{SNR}\right) & =\underset{\theta <0}{\sup}\frac{1}{L}\sum_{\ell =n_{t}}^{L-1}\{\theta \left(n_{r}+\frac{\mathrm{SNR}}{n_{t}}E\left[{∥E_{\ell }^{\left(T\right)}∥}_{F}^{2}\right]\right)+E\left[\log\det\left(I_{n_{r}}-\theta \frac{\mathrm{SNR}}{n_{t}}{\hat{H}}_{\ell }^{\left(T\right)}{\hat{H}}_{\ell }^{†(T)}\right)\right]\\ \; & \;\;\;\;\;-E\left[\theta Y_{\ell }^{†}{\left(I_{n_{r}}-\theta \frac{\mathrm{SNR}}{n_{t}}{\hat{H}}_{\ell }^{\left(T\right)}{\hat{H}}_{\ell }^{†(T)}\right)}^{-1}Y_{\ell }\right]\}. \end{array}$$

Following the steps used in ([29], Appendix D), it can be shown that, for θ<0,

(100) $$E\left[\theta Y_{\ell }^{†}{\left(I_{n_{r}}-\theta \frac{\mathrm{SNR}}{n_{t}}{\hat{H}}_{\ell }^{\left(T\right)}{\hat{H}}_{\ell }^{†(T)}\right)}^{-1}Y_{\ell }\right]\leq 0.$$

As observed in ([29], Appendix D), a good lower bound on $I_{T}^{\mathrm{gmi}}\left(\mathrm{SNR}\right)$ for high SNR follows by choosing

(101) $$\theta =\frac{-1}{n_{r}+\mathrm{SNR}\;n_{r}\epsilon_{*,T}^{2}}$$

where

(102) $$\epsilon_{*,T}^{2}=\max_{\begin{array}{c} r=1,\cdots ,n_{r},\\ t=1,\cdots ,n_{t},\\ \ell =n_{t},\cdots ,L-1 \end{array}}\;\epsilon_{\ell ,T}^{2}\left(r,t\right).$$

Hence, substituting the choice of θ in (101), and applying (100) to the RHS of (99), we obtain the following lower bound on $I_{T}^{\mathrm{gmi}}\left(\mathrm{SNR}\right)$:

(103) $$I_{T}^{\mathrm{gmi}}\left(\mathrm{SNR}\right)\geq \frac{1}{L}\sum_{\ell =n_{t}}^{L-1}\left\{E\left[\log\det\left(I_{n_{r}}+\frac{\mathrm{SNR}}{n_{t}n_{r}+n_{t}n_{r}\mathrm{SNR}\epsilon_{*,T}^{2}}{\hat{H}}_{\ell }^{\left(T\right)}{\hat{H}}_{\ell }^{†(T)}\right)\right]-1\right\}.$$

#### 5.2.2. $I_{T}^{\mathrm{gmi}}\left(\mathrm{SNR}\right)$ as T→∞

We next analyze the RHS of (103) in the limit as *T* tends to infinity. To this end, we note that, for $L\leq \frac{1}{2\lambda_{D}}$, the variance of the interpolation error tends to (15), namely

(104) $$\epsilon_{\ell }^{2}\left(t\right)=1-\int_{-1/2}^{1/2}\frac{\mathrm{SNR}{\left[f_{H}\left(\lambda \right)\right]}^{2}}{\mathrm{SNR}f_{H}\left(\lambda \right)+Ln_{t}}d\lambda$$

as *T* tends to infinity, irrespective of *ℓ* and *t*. We shall therefore omit the subscript and argument and write $\epsilon^{2}$ instead of $\epsilon_{\ell }^{2}\left(t\right)$. Please note that for a fixed *T*, the entries of

(105) $$\frac{1}{\sqrt{n_{t}n_{r}+n_{t}n_{r}\mathrm{SNR}\epsilon_{*,T}^{2}}}{\hat{H}}_{\ell }^{\left(T\right)}$$

are independent but not i.i.d., which follows from Part 2) of Lemma 1. However, as *T* tends to infinity, their distribution becomes identical due to (104) and hence they converge in distribution to

(106) $$\frac{1}{\sqrt{n_{t}n_{r}+n_{t}n_{r}\mathrm{SNR}\epsilon_{*,T}^{2}}}{\hat{H}}_{\ell }^{\left(T\right)}\;\overset{d}{⟶}\;\frac{1}{\sqrt{n_{t}n_{r}+n_{t}n_{r}\mathrm{SNR}\epsilon^{2}}}\bar{H}$$

where the entries of $\bar{H}$ are i.i.d. complex-Gaussian random variables with zero mean and variance $\left(1-\epsilon^{2}\right)$.

Next note that

(107) $$\log\det\left(I_{n_{r}}+\frac{\mathrm{SNR}}{n_{t}n_{r}+n_{t}n_{r}\mathrm{SNR}\epsilon_{*,T}^{2}}{\hat{H}}_{\ell }^{\left(T\right)}{\hat{H}}_{\ell }^{†(T)}\right)$$

is a nonnegative, continuous function with respect to the entries of the matrix

(108) $$\frac{1}{n_{t}n_{r}+n_{t}n_{r}\mathrm{SNR}\epsilon_{*,T}^{2}}{\hat{H}}_{\ell }^{\left(T\right)}{\hat{H}}_{\ell }^{†(T)}.$$

It therefore follows from Portmanteau’s lemma [30] that, as T→∞, the RHS of (103) can be lower-bounded by

$$\begin{array}{cc} \; & \lim_{T\to \infty }\;\frac{1}{L}\sum_{\ell =n_{t}}^{L-1}\left\{E\left[\log\det\left(I_{n_{r}}+\frac{\mathrm{SNR}}{n_{t}n_{r}+n_{t}n_{r}\mathrm{SNR}\epsilon_{*,T}^{2}}{\hat{H}}_{\ell }^{\left(T\right)}{\hat{H}}_{\ell }^{†(T)}\right)\right]-1\right\}\\ (109)\; & \;\geq \frac{L-n_{t}}{L}\left\{E\left[\log\det\left(I_{n_{r}}+\frac{\mathrm{SNR}}{n_{t}n_{r}+n_{t}n_{r}\mathrm{SNR}\epsilon^{2}}\bar{H}{\bar{H}}^{†}\right)\right]-1\right\}\\ (110)\; & \;\geq \frac{L-n_{t}}{L}\left(E\left[\log\det\left(\frac{\mathrm{SNR}}{n_{t}n_{r}+n_{t}n_{r}\mathrm{SNR}\epsilon^{2}}\bar{H}{\bar{H}}^{†}\right)\right]-1\right) \end{array}$$

where the last inequality follows from the lower bound $\log\det\left(I+A\right)\geq \log\det\;A$.

Combining (110) with (103), and using that, by assumption, $n_{t}=n_{r}$, we obtain that

$$\begin{array}{ccc} (111) & I^{\mathrm{gmi}}\left(\mathrm{SNR}\right) & ≜\lim_{T\to \infty }I_{T}^{\mathrm{gmi}}\left(\mathrm{SNR}\right)\\ (112) & \; & \geq \frac{L-n_{t}}{L}\left(n_{t}\log\mathrm{SNR}-n_{t}\log\left({n_{t}}^{2}+{n_{t}}^{2}\mathrm{SNR}\epsilon^{2}\right)+E\left[\log\det\;\bar{H}{\bar{H}}^{†}\right]-1\right). \end{array}$$

#### 5.2.3. The Pre-Log

It remains to compute a lower bound on the pre-log. To this end, we compute the limiting ratio of the RHS of (112) to logSNR as SNR tends to infinity. We first consider

$$\begin{array}{ccc} (113) & \mathrm{SNR}\;\epsilon^{2} & =\mathrm{SNR}\left(1-\int_{-1/2}^{1/2}\frac{\mathrm{SNR}{\left[f_{H}\left(\lambda \right)\right]}^{2}}{\mathrm{SNR}f_{H}\left(\lambda \right)+Ln_{t}}d\lambda \right)\\ (114)\; & & =\int_{-1/2}^{1/2}\frac{\mathrm{SNR}f_{H}\left(\lambda \right)Ln_{t}}{\mathrm{SNR}f_{H}\left(\lambda \right)+Ln_{t}}d\lambda . \end{array}$$

Since the integrand is bounded by

(115) $$0\leq \frac{\mathrm{SNR}f_{H}\left(\lambda \right)Ln_{t}}{\mathrm{SNR}f_{H}\left(\lambda \right)+L}\leq Ln_{t}$$

it follows that $0\leq \mathrm{SNR}\;\epsilon^{2}\leq Ln_{t}$, which implies that

(116) $$\lim_{\mathrm{SNR}\to \infty }\frac{\log\left({n_{t}}^{2}+{n_{t}}^{2}\mathrm{SNR}\;\epsilon^{2}\right)}{\log\mathrm{SNR}}=0.$$

We next consider the term $E\left[\log\det\;\bar{H}{\bar{H}}^{†}\right]-1$. Please note that by ([31], Lemma A.2) and the assumption $n_{t}=n_{r}$, we have

(117) $$\begin{array}{cc} E\left[\log\det\;\bar{H}{\bar{H}}^{†}\right]-1 & =n_{t}\log\left(1-\epsilon^{2}\right)+\sum_{b=0}^{n_{t}-1}\psi \left(n_{t}-b\right)-1 \end{array}$$

where ψ(·) is Euler’s digamma function [32]. Furthermore, since

(118) $$0\leq \frac{\mathrm{SNR}{\left[f_{H}\left(\lambda \right)\right]}^{2}}{\mathrm{SNR}f_{H}\left(\lambda \right)+Ln_{t}}\leq f_{H}\left(\lambda \right)$$

we have by the Dominated Convergence Theorem [28] that

(119) $$\lim_{\mathrm{SNR}\to \infty }\epsilon^{2}=\lim_{\mathrm{SNR}\to \infty }\left(1-\int_{-1/2}^{1/2}\frac{\mathrm{SNR}{\left[f_{H}\left(\lambda \right)\right]}^{2}}{\mathrm{SNR}f_{H}\left(\lambda \right)+Ln_{t}}d\lambda \right)=0$$

so $\log(1-\epsilon^{2})$ vanishes as the SNR tends to infinity. Combining (119) with (117) yields

(120) $$\lim_{\mathrm{SNR}\to \infty }\;\frac{E\left[\log\det\;\bar{H}{\bar{H}}^{†}\right]-1}{\log\mathrm{SNR}}=0.$$

It follows from (112), (116), and (120) that

$$\begin{array}{ccc} (121) & \Pi_{R^{*}} & \geq n_{t}\left(1-\frac{n_{t}}{L}\right)\\ (122)\; & & =\min\left(n_{t},n_{r}\right)\left(1-\frac{\min(n_{t},n_{r})}{L}\right),\;L\leq \frac{1}{2\lambda_{D}} \end{array}$$

where we have used that $n_{t}=n_{r}=\min\left(n_{t},n_{r}\right)$. Please note that the condition $L\leq \frac{1}{2\lambda_{D}}$ is necessary since otherwise (104) would not hold. This proves Theorem 1.

### 5.3. A Note on the Input Distribution

The pre-log in Theorem 1 is derived using codebooks whose entries are drawn i.i.d. from an $n_{t}$-variate Gaussian distribution with zero mean and identity covariance matrix. However, Gaussian inputs are not necessary to achieve the pre-log (25). In fact, as we shall argue next, the pre-log (25) can be achieved by any i.i.d. inputs with a probability density function satisfying $E[∥\bar{X}{∥}^{2}]\leq n_{t}$ and (26) and (27), namely,

$$\begin{array}{cc} (123)\; & p_{\bar{X}}\left(\bar{x}\right)\leq \frac{K}{\pi^{n_{t}}}e^{-∥\bar{x}{∥}^{2}},\;\bar{x}\in^{n_{t}}\\ (124)\; & \lim_{\mathrm{SNR}\to \infty }\;\frac{\log K}{\log\mathrm{SNR}}=0. \end{array}$$

Indeed, since the inputs have a density, they also satisfy $E[∥\bar{X}{∥}^{2}]>0$. To show that the conditions (26) and (27) suffice to achieve (25), we follow the steps in Section 5.2 but with F(SNR) replaced by

(125) $$F\left(\mathrm{SNR}\right)=n_{r}+\frac{\mathrm{SNR}}{\left(L-n_{t}\right)n_{t}}\sum_{\ell =n_{t}}^{L-1}E\left[{∥E_{\ell }^{\left(T\right)}{\bar{X}}_{\ell }∥}_{F}^{2}\right].$$

We then upper-bound F(SNR) and κ(θ,SNR) as follows. Using that for any two matrices A and B we have ${∥AB∥}_{F}^{2}\leq {∥A∥}_{F}^{2}\cdot {∥B∥}_{F}^{2}$ ([33], Section 5.6), and using that $E_{\ell }^{\left(T\right)}$ and ${\bar{X}}_{\ell }$ are independent, we can upper-bound F(SNR) by

(126) $$\begin{array}{c} F\left(\mathrm{SNR}\right)\leq n_{r}+\frac{\mathrm{SNR}}{\left(L-n_{t}\right)n_{t}}\sum_{\ell =n_{t}}^{L-1}E\left[{∥E_{\ell }^{\left(T\right)}∥}_{F}^{2}\right]\cdot E\left[{∥{\bar{X}}_{\ell }∥}^{2}\right]. \end{array}$$

As for κ(θ,SNR), we have

$$\begin{array}{cc} \; & E\left[\left.\exp\left(\frac{\theta }{n}D_{k}\left(m^{\prime }\right)\right)\right|y_{k},{\hat{H}}_{k}^{\left(T\right)}\right]\\ (127)\; & \;=\int_{{\bar{x}}_{k}}p_{\bar{X}}\left({\bar{x}}_{k}\right)\;\exp\left(\frac{\theta }{n}{∥y_{k}-\sqrt{\frac{\mathrm{SNR}}{n_{t}}}\;{\hat{H}}_{k}^{\left(T\right)}{\bar{x}}_{k}∥}^{2}\right)d{\bar{x}}_{k}\\ (128)\; & \;\leq \int_{{\bar{x}}_{k}}\frac{K}{\pi^{n_{t}}}\;\exp\left(-∥{\bar{x}}_{k}{∥}^{2}+\frac{\theta }{n}{∥y_{k}-\sqrt{\frac{\mathrm{SNR}}{n_{t}}}\;{\hat{H}}_{k}^{\left(T\right)}{\bar{x}}_{k}∥}^{2}\right)d{\bar{x}}_{k}\\ (129)\; & \;=\frac{K}{\det\left(I_{n_{r}}-\frac{\theta }{n}\frac{\mathrm{SNR}}{n_{t}}{\hat{H}}_{k}^{\left(T\right)}{\hat{H}}_{k}^{†(T)}\right)}\exp\left(\frac{\theta }{n}y_{k}^{†}{\left(I_{n_{r}}-\frac{\theta }{n}\frac{\mathrm{SNR}}{n_{t}}{\hat{H}}_{k}^{\left(T\right)}{\hat{H}}_{k}^{†(T)}\right)}^{-1}y_{k}\right). \end{array}$$

Here (128) follows from (123), and (129) follows by evaluating the integral as in ([17], Appendix A). By following the steps in Section 5.2, and by choosing

(130) $$\theta =\frac{-1}{n_{r}+\mathrm{SNR}\;n_{r}\epsilon_{*,T}^{2}E\left[∥\bar{X}{∥}^{2}\right]}$$

where $\epsilon_{*,T}^{2}$ is given in (102), we obtain from (126) and (129) that

(131) $$\begin{array}{cc} I_{T}^{\mathrm{gmi}}\left(\mathrm{SNR}\right) & \geq \frac{1}{L}\sum_{\ell =n_{t}}^{L-1}\left\{E\left[\log\det\left(I_{n_{r}}+\frac{\mathrm{SNR}}{n_{t}n_{r}+n_{t}n_{r}\mathrm{SNR}\epsilon_{*,T}^{2}E\left[∥\bar{X}{∥}^{2}\right]}{\hat{H}}_{\ell }^{\left(T\right)}{\hat{H}}_{\ell }^{†(T)}\right)\right]\right\}\\ \; & \;-\frac{L-n_{t}}{L}\left(1+\log K\right). \end{array}$$

Taking the limit as *T* tends to infinity, and repeating the steps in Section 5.2, it follows that

$$\begin{array}{ccc} (132) & I^{\mathrm{gmi}}\left(\mathrm{SNR}\right) & =\lim_{T\to \infty }I_{T}^{\mathrm{gmi}}\left(\mathrm{SNR}\right)\\ (133) & \; & \geq \frac{L-n_{t}}{L}\left(E\left[\log\;\det\left(\frac{\mathrm{SNR}}{n_{t}n_{r}+n_{t}n_{r}\mathrm{SNR}\;\epsilon^{2}E\left[∥\bar{X}{∥}^{2}\right]}\bar{H}{\bar{H}}^{†}\right)\right]-1-\log K\right)\\ \; & & =\frac{L-n_{t}}{L}(n_{t}\log\mathrm{SNR}-n_{t}\log\left({n_{t}}^{2}+{n_{t}}^{2}\mathrm{SNR}\;\epsilon^{2}E\left[∥\bar{X}{∥}^{2}\right]\right)\\ (133) & \; & \;\;\;\;\;\;\;\;+\;E\left[\log\det\;\bar{H}{\bar{H}}^{†}\right]-1-\log K) \end{array}$$

where we have again used the assumption $n_{t}=n_{r}$.

We conclude by evaluating the limiting ratio of the RHS of (134) to logSNR as SNR tends to infinity. Using (115) and that $E[∥\bar{X}{∥}^{2}]\leq n_{t}$, we obtain that

(135) $$\lim_{\mathrm{SNR}\to \infty }\frac{\log\left({n_{t}}^{2}+{n_{t}}^{2}\mathrm{SNR}\;\epsilon^{2}E\left[∥\bar{X}{∥}^{2}\right]\right)}{\log\mathrm{SNR}}=0.$$

This in turn yields together with (120) that

(136) $$\lim_{\mathrm{SNR}\to \infty }\frac{I^{\mathrm{gmi}}\left(\mathrm{SNR}\right)}{\log\mathrm{SNR}}\geq n_{t}\left(1-\frac{n_{t}}{L}\right)$$

provided that

(137) $$\begin{array}{c} \lim_{\mathrm{SNR}\to \infty }\;\frac{\log K}{\log\mathrm{SNR}}=0. \end{array}$$

It thus follows that any i.i.d. input distribution satisfying $E[∥\bar{X}{∥}^{2}]\leq n_{t}$ and (26) and (27) achieves the pre-log (25).

## 6. Proof of Theorem 2

In contrast to the proof of Theorem 1, for the fading MAC, it is not sufficient to restrict ourselves to the case of $n_{t,1}=n_{t,2}=n_{r}$. For example, increasing $n_{r}$ beyond $n_{t,1}$ and $n_{t,2}$ does not increase the single-rate pre-logs $\Pi_{R_{1}^{*}}$ and $\Pi_{R_{2}^{*}}$, but it does increase the pre-log of the achievable sum-rate $\Pi_{R_{1+2}^{*}}$. For the proof of Theorem 2, we therefore consider a general setup of $n_{t,1}$, $n_{t,2}$, and $n_{r}$.

We derive the achievable pre-logs for the MAC case by following similar steps as in the point-to-point case. We first consider the average error probability, averaged over the ensemble of i.i.d. Gaussian codebooks. Let ${\bar{P}}_{e}$ and ${\bar{P}}_{e}\left(m_{1},m_{2}\right)$ be the ensemble-average error probability and the ensemble-average error probability when messages $m_{1}$ and $m_{2}$ are transmitted, respectively. Due to the symmetry of the codebook construction, ${\bar{P}}_{e}$ is equal to ${\bar{P}}_{e}\left(1,1\right)$ and it therefore suffices to consider ${\bar{P}}_{e}\left(1,1\right)$ to derive the achievable rates. Let $E(m_{1}^{\prime },m_{2}^{\prime })$ denote the event that $D\left(m_{1}^{\prime },m_{2}^{\prime }\right)\leq D\left(1,1\right)$, where D(·,·) was defined in (45). Using the union bound, the error probability ${\bar{P}}_{e}\left(1,1\right)$ can be upper-bounded as

$$\begin{array}{ccc} (138) & {\bar{P}}_{e}\left(1,1\right) & =\mathrm{Pr}\left\{\underset{\left(m_{1}^{\prime },m_{2}^{\prime }\right)\neq \left(1,1\right)}{⋃}E\left(m_{1}^{\prime },m_{2}^{\prime }\right)\right\}\\ (139)\; & & \;\;\;\;\;\leq \mathrm{Pr}\left\{\underset{m_{1}^{\prime }\neq 1}{⋃}E\left(m_{1}^{\prime },1\right)\right\}+\mathrm{Pr}\left\{\underset{m_{2}^{\prime }\neq 1}{⋃}E\left(1,m_{2}^{\prime }\right)\right\}+\mathrm{Pr}\left\{\underset{m_{1}^{\prime }\neq 1}{⋃}\underset{m_{2}^{\prime }\neq 1}{⋃}E\left(m_{1}^{\prime },m_{2}^{\prime }\right)\right\}. \end{array}$$

We next analyze the three probabilities on the RHS of (139). Let the matrix $E_{s,k}^{\left(T\right)}$, s=1,2 with entries $E_{s,k}^{\left(T\right)}\left(r,t\right)$ be the estimation-error matrix in estimating $H_{s,k}$, i.e.,

(140) $$E_{s,k}^{\left(T\right)}=H_{s,k}-{\hat{H}}_{s,k}^{\left(T\right)}.$$

To facilitate the analysis, we first generalize F(SNR) and $T_{\delta }$, defined in the point-to-point case in (77) and (78), to the MAC case:

$$\begin{array}{ccc} (141) & F(\mathrm{SNR}) & ≜n_{r}+\frac{\mathrm{SNR}}{L-n_{t,1}-n_{t,2}}\sum_{\ell =n_{t,1}+n_{t,2}}^{L-1}E\left[{∥E_{1,\ell }^{\left(T\right)}∥}_{F}^{2}+{∥E_{2,\ell }^{\left(T\right)}∥}_{F}^{2}\right],\\ & T_{\delta } & ≜\{\left(x_{s,k},y_{k},{\hat{H}}_{s,k}^{\left(T\right)}\right),\;k=0,\cdots ,n^{\prime }-1,\;s=1,2:\\ (142)\; & & \;\;\;\left|\frac{1}{n}\sum_{k\in D^{\left(n^{\prime }\right)}}{∥y_{k}-\sqrt{\mathrm{SNR}}\;{\hat{H}}_{1,k}^{\left(T\right)}x_{1,k}-\sqrt{\mathrm{SNR}}\;{\hat{H}}_{2,k}^{\left(T\right)}x_{2,k}∥}^{2}-F\left(\mathrm{SNR}\right)\right|<\delta \} \end{array}$$

for some δ>0, with $n^{\prime }$ given in (41) and $D^{\left(n^{\prime }\right)}=\left\{0,\cdots ,n^{\prime }-1\right\}\cap D$. Using F(SNR) and the typical set $T_{\delta }$, we continue by evaluating the GMI for each of the three probabilities on the RHS of (139), which correspond to the error events $\left(m_{1}^{\prime }\neq 1,m_{2}^{\prime }=1\right)$, $\left(m_{1}^{\prime }=1,m_{2}^{\prime }\neq 1\right)$, and $\left(m_{1}^{\prime }\neq 1,m_{2}^{\prime }\neq 1\right)$.

### 6.1. Error Event $\left(m_{1}^{\prime }\neq 1,m_{2}^{\prime }=1\right)$

Following the steps in Section 5.2 to derive (86), we can upper-bound the ensemble-average error probability for the error event $E(m_{1}^{\prime },1)$, $m_{1}^{\prime }\neq 1$ as

(143) $$\begin{array}{cc} \; & \mathrm{Pr}\left\{\underset{m_{1}^{\prime }\neq 1}{⋃}E\left(m_{1}^{\prime },1\right)\right\}\\ \; & \;\leq e^{nR_{1}}\cdot \mathrm{Pr}\left\{\left.\frac{1}{n}\cdot D\left(m_{1}^{\prime },1\right)<F\left(\mathrm{SNR}\right)+\delta \;\right|\left\{\left(X_{s}^{n^{\prime }}\left(1\right),Y^{n^{\prime }},{\hat{H}}_{s}^{\left(T\right),n^{\prime }}\right),s=1,2\right\}\in T_{\delta }\right\}\\ \; & \;+\mathrm{Pr}\left\{\left\{\left(X_{s}^{n^{\prime }}\left(1\right),Y^{n^{\prime }},{\hat{H}}_{s}^{\left(T\right),n^{\prime }}\right),s=1,2\right\}\in T_{\delta }^{c}\right\},\;m_{1}^{\prime }\neq 1. \end{array}$$

Note that the second probability on the RHS of (143) vanishes as *n* tends to infinity, which can be shown along the lines of the proof of Lemma 2.

The GMI of user 1 gives the rate of exponential decay of the term

(144) $$\mathrm{Pr}\left\{\left.\frac{1}{n}\cdot D\left(m_{1}^{\prime },1\right)<F\left(\mathrm{SNR}\right)+\delta \right|\left\{\left(X_{s}^{n^{\prime }}\left(1\right),Y^{n^{\prime }},{\hat{H}}_{s}^{\left(T\right),n^{\prime }}\right),s=1,2\right\}\in T_{\delta }\right\}$$

as n→∞. Its evaluation requires the expression of the log moment-generating function of the metric $D(m_{1}^{\prime },1)$, conditioned on the channel outputs, on $m_{2}^{\prime }=1$, and on the fading estimates, which is defined as

(145) $$\begin{array}{cc} \kappa_{1,n} & \left(\theta ,y^{n^{\prime }},x_{2}^{n^{\prime }}\left(1\right),{\hat{H}}_{1}^{\left(T\right),n^{\prime }},{\hat{H}}_{2}^{\left(T\right),n^{\prime }}\right)\\ \; & \;≜\log E\left[\exp\left(\frac{\theta }{n}\sum_{k\in D^{\left(n^{\prime }\right)}}D_{k}\left(m_{1}^{\prime },1\right)\right)|\left\{\left(y_{k},x_{2,k}\left(1\right),{\hat{H}}_{1,k}^{\left(T\right)},{\hat{H}}_{2,k}^{\left(T\right)}\right),\;k\in D^{\left(n^{\prime }\right)}\right\}\right] \end{array}$$

where

(146) $$D_{k}\left(m_{1}^{\prime },m_{2}^{\prime }\right)≜{∥y_{k}-\sqrt{\mathrm{SNR}}\;{\hat{H}}_{1,k}^{\left(T\right)}x_{1,k}\left(m_{1}^{\prime }\right)-\sqrt{\mathrm{SNR}}\;{\hat{H}}_{2,k}^{\left(T\right)}x_{2,k}\left(m_{2}^{\prime }\right)∥}^{2}.$$

Following the steps used in Section 5.2 to obtain (90) and (91), it can be shown that

(147) $$\begin{array}{cc} \; & \kappa_{1,n}\left(\theta ,y^{n^{\prime }},x_{2}^{n^{\prime }}\left(1\right),{\hat{H}}_{1}^{\left(T\right),n^{\prime }},{\hat{H}}_{2}^{\left(T\right),n^{\prime }}\right)\\ \; & \;=\sum_{k\in D^{\left(n^{\prime }\right)}}\{\frac{\theta }{n}{\left(y_{k}-\sqrt{\mathrm{SNR}}\;{\hat{H}}_{2,k}^{\left(T\right)}x_{2,k}\left(1\right)\right)}^{†}{\left(I_{n_{r}}-\frac{\theta }{n}\mathrm{SNR}\;{\hat{H}}_{1,k}^{\left(T\right)}{\hat{H}}_{1,k}^{†(T)}\right)}^{-1}\left(y_{k}-\sqrt{\mathrm{SNR}}\;{\hat{H}}_{2,k}^{\left(T\right)}x_{2,k}\left(1\right)\right)\\ \; & \;\;\;\;\;\;\;\;\;\;-\log\det\left(I_{n_{r}}-\frac{\theta }{n}\mathrm{SNR}\;{\hat{H}}_{1,k}^{\left(T\right)}{\hat{H}}_{1,k}^{†(T)}\right)\}. \end{array}$$

Then, following the steps used in Section 5.2 to derive (92)–(94), we obtain that, for all θ<0,

$$\begin{array}{cc} \; & \lim_{n\to \infty }\frac{1}{n}\cdot \kappa_{1,n}\left(n\theta ,y^{n^{\prime }},x_{2}^{n^{\prime }}\left(1\right),{\hat{H}}_{1}^{\left(T\right),n^{\prime }},{\hat{H}}_{2}^{\left(T\right),n^{\prime }}\right)\\ (148)\; & \;=\frac{1}{L-n_{t,1}-n_{t,2}}\sum_{\ell =n_{t,1}+n_{t,2}}^{L-1}\left(g_{1,\ell }\left(\theta ,\mathrm{SNR}\right)-E\left[\log\det\left(I_{n_{r}}-\theta \;\mathrm{SNR}\;{\hat{H}}_{1,\ell }^{\left(T\right)}{\hat{H}}_{1,\ell }^{†(T)}\right)\right]\right)\\ (149)\; & \;≜\kappa_{1}\left(\theta ,\mathrm{SNR}\right),\;\mathrm{almost}\;\mathrm{surely} \end{array}$$

where the last step should be regarded as the definition of $\kappa_{1}\left(\theta ,\mathrm{SNR}\right)$. In (148), we define

(150) $$\begin{array}{cc} g_{1,\ell }\left(\theta ,\mathrm{SNR}\right) & ≜E\left[\theta {\left(Y_{\ell }-\sqrt{\mathrm{SNR}}\;{\hat{H}}_{2,\ell }^{\left(T\right)}X_{2,\ell }\right)}^{†}{\left(I_{n_{r}}-\theta \;\mathrm{SNR}\;{\hat{H}}_{1,\ell }^{\left(T\right)}{\hat{H}}_{1,\ell }^{†(T)}\right)}^{-1}\left(Y_{\ell }-\sqrt{\mathrm{SNR}}\;{\hat{H}}_{2,\ell }^{\left(T\right)}X_{2,\ell }\right)\right]. \end{array}$$

Following the derivation in [16,17], we can then upper-bound

(151) $$\mathrm{Pr}\left\{\underset{m_{1}^{\prime }\neq 1}{⋃}E\left(m_{1}^{\prime },1\right)\right\}\leq \exp\left(nR_{1}\right)\exp\left(-n\left(I_{1,T}^{\mathrm{gmi}}\left(\mathrm{SNR}\right)-\delta^{\prime }\right)\right)+\varepsilon_{1}\left(\delta^{\prime },n\right)$$

for any $\delta^{\prime }>0$, and for some $\varepsilon_{1}\left(\delta^{\prime },n\right)$ satisfying

(152) $$\lim_{n\to \infty }\varepsilon_{1}\left(\delta^{\prime },n\right)=0,\;\delta^{\prime }>0.$$

On the RHS of (151), $I_{1,T}^{\mathrm{gmi}}\left(\mathrm{SNR}\right)$ denotes the GMI for user 1 as a function of the SNR for a fixed *T*, i.e.,

(153) $$I_{1,T}^{\mathrm{gmi}}\left(\mathrm{SNR}\right)=\frac{L-n_{t,1}-n_{t,2}}{L}\left(\underset{\theta <0}{\sup}\;\left(\theta F\left(\mathrm{SNR}\right)-\kappa_{1}\left(\theta ,\mathrm{SNR}\right)\right)\right).$$

The pre-factor $(L-n_{t,1}-n_{t,2})/L$ equals the fraction of time used for data transmission. The bound (151) implies that, for all rates below $I_{1,T}^{\mathrm{gmi}}\left(\mathrm{SNR}\right)$, the error probability in decoding user 1’s message for the scheme described in Section 4 vanishes as *n* tends to infinity.

Combining (141) and (148) with (153), we obtain that

(154) $$\begin{array}{cc} I_{1,T}^{\mathrm{gmi}}\left(\mathrm{SNR}\right)=\underset{\theta <0}{\sup}\;\frac{1}{L}\sum_{\ell =n_{t,1}+n_{t,2}}^{L-1}\{\theta & \left(n_{r}+\mathrm{SNR}\;E\left[{∥E_{1,\ell }^{\left(T\right)}∥}_{F}^{2}+{∥E_{2,\ell }^{\left(T\right)}∥}_{F}^{2}\right]\right)-g_{1,\ell }\left(\theta ,\mathrm{SNR}\right)\\ \; & \;\;+E\left[\log\det\left(I_{n_{r}}-\theta \;\mathrm{SNR}\;{\hat{H}}_{1,\ell }^{\left(T\right)}{\hat{H}}_{1,\ell }^{†(T)}\right)\right]\}. \end{array}$$

Since the supremum over θ<0 is difficult to evaluate, we next consider a lower bound on $I_{1,T}^{\mathrm{gmi}}\left(\mathrm{SNR}\right)$. By noting that $g_{1,\ell }\left(\theta ,\mathrm{SNR}\right)\leq 0$ for θ≤0 (which can be shown using the technique developed in ([29], Appendix D), and by choosing

(155) $$\theta =\frac{-1}{n_{r}+n_{r}\left(n_{t,1}+n_{t,2}\right)\mathrm{SNR}\;\epsilon_{*,T}^{2}}$$

where

(156) $$\epsilon_{*,T}^{2}=\max_{\begin{array}{c} s=1,2,\\ r=1,\cdots ,n_{r},\\ t=1,\cdots ,n_{t,s},\\ \ell =n_{t,1}+n_{t,2},\cdots ,L-1 \end{array}}\;E\left[{\left|E_{s,\ell }^{\left(T\right)}\left(r,t\right)\right|}^{2}\right]$$

we obtain the following lower bound on $I_{1,T}^{\mathrm{gmi}}\left(\mathrm{SNR}\right)$:

(157) $$\begin{array}{c} I_{1,T}^{\mathrm{gmi}}\left(\mathrm{SNR}\right)\geq \frac{1}{L}\sum_{\ell =n_{t,1}+n_{t,2}}^{L-1}E\left[\log\det\left(I_{n_{r}}+\frac{\mathrm{SNR}\;{\hat{H}}_{1,\ell }^{\left(T\right)}{\hat{H}}_{1,\ell }^{†(T)}}{n_{r}+n_{r}\left(n_{t,1}+n_{t,2}\right)\mathrm{SNR}\;\epsilon_{*,T}^{2}}\right)-1\right]. \end{array}$$

(As pointed out in Section 5, this choice of θ yields a good lower bound at high SNR.) We continue by analyzing the RHS of (157) in the limit as the observation window *T* of the channel estimator tends to infinity. To this end, we note that, for $L\leq \frac{1}{2\lambda_{D}}$, the variance of the interpolation error tends to (15) (with SNR in (15) replaced by $n_{t}\mathrm{SNR}$ due to the difference between the point-to-point channel model (1) and the MAC channel model (40)), so

(158) $$\lim_{T\to \infty }E\left[{\left|E_{s,\ell }^{\left(T\right)}\left(r,t\right)\right|}^{2}\right]=\epsilon^{2}=1-\int_{-1/2}^{1/2}\frac{\mathrm{SNR}{\left[f_{H}\left(\lambda \right)\right]}^{2}}{\mathrm{SNR}f_{H}\left(\lambda \right)+L}d\lambda$$

irrespective of s,ℓ,r, and *t*. It follows that the estimate ${\hat{H}}_{1,\ell }^{\left(T\right)}$ tends to ${\bar{H}}_{1}$ in distribution as *T* tends to infinity, which implies that

(159) $$\begin{array}{c} \frac{{\hat{H}}_{1,\ell }^{\left(T\right)}{\hat{H}}_{1,\ell }^{†(T)}}{n_{r}+n_{r}\left(n_{t,1}+n_{t,2}\right)\mathrm{SNR}\;\epsilon_{*,T}^{2}}\;\overset{d}{⟶}\;\frac{{\bar{H}}_{1}{\bar{H}}_{1}^{†}}{n_{r}+n_{r}\left(n_{t,1}+n_{t,2}\right)\mathrm{SNR}\;\epsilon^{2}} \end{array}$$

where the $n_{r}\times n_{t,1}$ entries of ${\bar{H}}_{1}$ are i.i.d., circularly-symmetric, complex-Gaussian random variables with zero mean and variance $\left(1-\epsilon^{2}\right)$. Using Portmanteau’s lemma (as used in (109)), we obtain that

$$\begin{array}{ccc} (160) & I_{1}^{\mathrm{gmi}}\left(\mathrm{SNR}\right) & =\lim_{T\to \infty }I_{1,T}^{\mathrm{gmi}}\left(\mathrm{SNR}\right)\\ (161)\; & & \geq \frac{L-n_{t,1}-n_{t,2}}{L}\left(E\left[\log\det\left(I_{n_{r}}+\frac{\mathrm{SNR}\;{\bar{H}}_{1}{\bar{H}}_{1}^{†}}{n_{r}+n_{r}\left(n_{t,1}+n_{t,2}\right)\mathrm{SNR}\;\epsilon^{2}}\right)\right]-1\right)\\ \; & & \geq \frac{L-n_{t,1}-n_{t,2}}{L}\min\left(n_{r},n_{t,1}\right)\left[\log\mathrm{SNR}-\log\left(n_{r}+n_{r}\left(n_{t,1}+n_{t,2}\right)\;\mathrm{SNR}\;\epsilon^{2}\right)\right]\\ (162)\; & & \;+\frac{L-n_{t,1}-n_{t,2}}{L}\Psi_{1} \end{array}$$

where

(163) $$\Psi_{1}≜\left\{\begin{array}{cc} E\left[\log\det\;{\bar{H}}_{1}{\bar{H}}_{1}^{†}\right]-1, & n_{r}\leq n_{t,1}\\ E\left[\log\det\;{\bar{H}}_{1}^{†}{\bar{H}}_{1}\right]-1, & n_{r}>n_{t,1}. \end{array}\right.$$

The inequality (162) follows by lower-bounding $\log\det\left(I+A\right)\geq \log\det A$.

By evaluating the limiting ratio of the RHS of (162) to logSNR as SNR tends to infinity following similar steps as in Section 5.2.3, we obtain the following lower bound on the maximum achievable pre-log of user 1:

(164) $$\Pi_{R_{1}^{*}}\geq \min\left(n_{r},n_{t,1}\right)\left(1-\frac{n_{t,1}+n_{t,2}}{L}\right),\;L\leq \frac{1}{2\lambda_{D}}.$$

As in the point-to-point case, the condition $L\leq 1/(2\lambda_{D})$ is necessary to obtain (15). The lower bound (164) yields one boundary of the pre-log region presented in Theorem 2.

### 6.2. Error Event $\left(m_{1}^{\prime }=1,m_{2}^{\prime }\neq 1\right)$

The error event $\left(m_{1}^{\prime }=1,m_{2}^{\prime }\neq 1\right)$ can be analyzed by swapping user 1 and user 2 and then using the results obtained in the previous subsection for the error event $\left(m_{1}^{\prime }\neq 1,m_{2}^{\prime }=1\right)$. We thus have the lower bound

(165) $$\begin{array}{cc} \Pi_{R_{2}^{*}} & \geq \min\left(n_{r},n_{t,2}\right)\left(1-\frac{n_{t,1}+n_{t,2}}{L}\right),\;L\leq \frac{1}{2\lambda_{D}} \end{array}$$

which yields the second boundary of the pre-log region presented in Theorem 2.

### 6.3. Error Event $\left(m_{1}^{\prime }\neq 1,m_{2}^{\prime }\neq 1\right)$

For the error event $\left(m_{1}^{\prime }\neq 1,m_{2}^{\prime }\neq 1\right)$, the analysis of the achievable sum rate follows the same analysis as in the point-to-point case (Section 5.2). More specifically, the GMI $I_{1+2,T}^{\mathrm{gmi}}\left(\mathrm{SNR}\right)$ that describes the exponential decay of the term

(166) $$\mathrm{Pr}\left\{\left.\frac{1}{n}\cdot D\left(m_{1}^{\prime },m_{2}^{\prime }\right)<F\left(\mathrm{SNR}\right)+\delta \right|\left\{\left(X_{s}^{n^{\prime }}\left(1\right),Y^{n^{\prime }},{\hat{H}}_{s}^{\left(T\right),n^{\prime }}\right),s=1,2\right\}\in T_{\delta }\right\}$$

can be viewed as the GMI of an $n_{r}\times \left(n_{t,1}+n_{t,2}\right)$-dimensional point-to-point MIMO channel with fading matrix $\left[H_{1,k},H_{2,k}\right]$ and fading estimate matrix $\left[{\hat{H}}_{1,k}^{\left(T\right)},{\hat{H}}_{2,k}^{\left(T\right)}\right]$. The maximum achievable sum-rate pre-log can therefore be obtained by following the same steps as in Section 5.2, but with arbitrary $n_{r}$ and $n_{t}=n_{t,1}+n_{t,2}$. It can thus be shown that the maximum achievable sum-rate pre-log $\Pi_{R_{1+2}^{*}}$ is lower-bounded by

(167) $$\begin{array}{c} \Pi_{R_{1+2}^{*}}\geq \min\left(n_{r},n_{t,1}+n_{t,2}\right)\left(1-\frac{n_{t,1}+n_{t,2}}{L}\right),\;L\leq \frac{1}{2\lambda_{D}}. \end{array}$$

On the RHS of (167), the term $\min(n_{r},n_{t,1}+n_{t,2})$ corresponds to the MIMO gain, which is given by the minimum number of receive and transmit antennas, and the term $\left(1-\frac{n_{t,1}+n_{t,2}}{L}\right)$ corresponds to the fraction of time indices for data transmission. This yields the third boundary of the pre-log region presented in Theorem 2.

## 7. Conclusions

In this paper, we studied a communication scheme for MIMO fading channels that estimates the fading via transmission of pilot symbols at regular intervals and feeds the fading estimates to the nearest neighbor decoder. Restricting ourselves to fading processes with a bandlimited power spectral density, we studied the information rates achievable with this scheme at high SNR. Specifically, we analyzed the achievable rate pre-log, defined as the limiting ratio of the achievable rate to the logarithm of the SNR in the limit as the SNR tends to infinity.

We showed that in order to obtain fading estimates whose variance vanishes as the SNR tends to infinity, the portion of time required for pilot transmission must be greater than or equal to the number of transmit antennas times twice the bandwidth of the fading power spectral density. We demonstrated that in this case, the nearest neighbor decoder achieves the capacity pre-log of the coherent fading channel times the fraction of time used for the transmission of data. Hence, the loss with respect to the coherent case is solely due to the transmission of pilots used to obtain accurate fading estimates. Our achievability bounds are tight in the sense that any scheme using as many pilots as our proposed scheme cannot achieve a higher pre-log using a nearest neighbor decoder. Furthermore, if the inverse of twice the bandwidth of the fading process is an integer, then, for MISO channels, our scheme achieves the capacity pre-log of the noncoherent fading channel derived by Koch and Lapidoth [11]. For noncoherent MIMO channels, our scheme achieves the best so far known lower bound on the capacity pre-log obtained by Etkin and Tse [12]. Since the last result only yields a lower bound on the capacity pre-log of MIMO channels, there may exist other schemes achieving a better pre-log than our scheme.

## Figures and Tables

**Table 1:** Typical values of $L^{*}$ for various environments with $f_{c}$ ranging from 800 MHz to 5 GHz. The values of the delay spread are taken from [12,25] for indoor and urban environments and from [26] for hilly area environments.

Environment	Delay Spread $\sigma_{\tau }$	Mobile Speed *v*	$\lambda_{D}\approx 5\sigma_{\tau }\frac{v}{c}f_{c}$	$L^{*}$
Indoor	10–100 ns	5 km/h	$2\times 10^{-7}$–$10^{-5}$	$5\times 10^{4}$–$2.5\times 10^{6}$
Urban	1–2 μs	5 km/h	$2\times 10^{-5}$–$2\times 10^{-4}$	$2.5\times 10^{3}$–$2.5\times 10^{4}$
Urban	1–2 μs	75 km/h	$2\times 10^{-4}$–0.004	125–$2.5\times 10^{3}$
Hilly area	3–10 μs	200 km/h	0.002–0.05	10–250

## Appendix A. Proof of Lemma 1

We prove each part of Lemma 1 in a separate item:

1. By the orthogonality principle [34], it follows that ${\hat{H}}_{k}^{\left(T\right)}\left(r,t\right)$ and $E_{k}^{\left(T\right)}\left(r,t\right)$ are uncorrelated. Noting that the pilot symbols are unity, we can write (72) as
  (A1) $${\hat{H}}_{k}^{\left(T\right)}\left(r,t\right)=\sum_{\begin{array}{c} k^{\prime }=k-TL:\\ k^{\prime }\in P \end{array}}^{k+TL}a_{k^{\prime }}\left(r,t\right)\left(\sqrt{\frac{\mathrm{SNR}}{n_{t}}}H_{k^{\prime }}\left(r,t\right)+Z_{k^{\prime }}\left(r\right)\right),\;k\in D.$$
  Since the processes $\left\{H_{k}\left(r,t\right),\;k\in \right\}$ and $\left\{Z_{k}\left(r\right),\;k\in \right\}$ are zero-mean complex-Gaussian, we have from (A1) and the orthogonality principle that ${\hat{H}}_{k}^{\left(T\right)}\left(r,t\right)$ and $E_{k}^{\left(T\right)}\left(r,t\right)$ are independent zero-mean complex-Gaussian random variables.
2. Recall from Section 5.1 that the time index *k* can be written as k=jL+ℓ. Then, for k∈D, we have $\ell =n_{t},\cdots ,L-1$, and for k∈P we have $\ell =0,\cdots ,n_{t}-1$. Since the pilot vectors are transmitted sequentially from $p_{1}$ to $p_{n_{t}}$, we have for (jL+ℓ)∈P that
  (A2) $$x_{jL+\ell }=p_{\ell +1},\;\ell =0,\cdots ,n_{t}-1.$$
  That is, the pilot vectors that help estimate the fading coefficients from transmit antenna *t* are transmitted at time instants whose remainder after division by *L* is equal to t−1. This implies that in order to estimate $H_{k}\left(r,t\right)$, there is no loss in optimality by considering only the outputs $Y_{k^{\prime }}\left(r\right)$ for $k^{\prime }\in P\cap \left\{k-TL,\cdots ,k+TL\right\}$ satisfying
  (A3) $$k^{\prime }\;\mathrm{mod}\;L=t-1.$$
  Indeed, the channel outputs $Y_{k^{\prime }}\left(r\right)$, $k^{\prime }\;\mathrm{mod}\;L\neq t-1$ correspond to $H_{k^{\prime }}\left(r,t^{\prime }\right)$, $t^{\prime }\neq t$, which are independent from $H_{k}\left(r,t\right)$ since we assumed that the fading processes corresponding to different transmit and receive antennas are independent. It follows that for estimation at time k=jL+ℓ, the coefficients $a_{k^{\prime }}\left(r,t\right)$ that minimize the mean-squared error depend only on *L* and *ℓ* [23]. The fading estimate (72) can then be expressed as
  $$\begin{array}{ccc} (A4) & {\hat{H}}_{jL+\ell }^{\left(T\right)}\left(r,t\right) & =\sum_{\tau =-T}^{T-1}\alpha_{-\tau L,\ell }\left(r,t\right)Y_{(j-\tau )L+t-1}\left(r\right)\\ (A5)\; & & =\sum_{\tau =-T}^{T-1}\alpha_{-\tau L,\ell }\left(r,t\right)\left(\sqrt{\frac{\mathrm{SNR}}{n_{t}}}H_{(j-\tau )L+t-1}\left(r,t\right)+Z_{(j-\tau )L+t-1}\left(r\right)\right) \end{array}$$
  where for a given *L* and $\ell =n_{t},\cdots ,L-1$, we defined
  (A6) $$\alpha_{-\tau L,\ell }\left(r,t\right)≜a_{(j-\tau )L+t-1}\left(r,t\right),\;\tau =-T,\cdots ,T-1.$$
  Noting again that the $n_{r}\cdot n_{t}$ processes $\left\{H_{k}\left(r,t\right),\;k\in \right\}$ are independent from each other and have the same law, we obtain the following results from (A5):
  (a) For a given *t*, the time differences between the index of interest (jL+ℓ) and the positions of pilots ((j−τ)L+t−1) do not depend on *r*. It thus follows that for a given *t*, the optimal coefficients $\alpha_{-\tau L,\ell }\left(r,t\right)$ are identical for all $r=1,\cdots ,n_{r}$ [23]. This implies that for a given *t* and *ℓ*, the $n_{r}$ processes
    (A7) $$\left\{\left({\hat{H}}_{jL+\ell }^{\left(T\right)}\left(1,t\right),E_{jL+\ell }^{\left(T\right)}\left(1,t\right)\right),\;j\in \mathbb{Z}\right\},\cdots ,\left\{\left({\hat{H}}_{jL+\ell }^{\left(T\right)}\left(n_{r},t\right),E_{jL+\ell }^{\left(T\right)}\left(n_{r},t\right)\right),\;j\in \mathbb{Z}\right\}$$
    are independent and have the same law.
  (b) For a given *r*, the time differences between the index of interest (jL+ℓ) and the position of pilots ((j−τ)L+t−1) depend on *t*. It thus follows from [23] that for a given *r*, the optimal coefficients $\alpha_{-\tau L,\ell }\left(r,t\right)$ are generally different for $t=1,\cdots ,n_{t}$. This implies that for a given *r* and *ℓ*, the $n_{t}$ processes
    (A8) $$\left\{\left({\hat{H}}_{jL+\ell }^{\left(T\right)}\left(r,1\right),E_{jL+\ell }^{\left(T\right)}\left(r,1\right)\right),\;j\in \mathbb{Z}\right\},\cdots ,\left\{\left({\hat{H}}_{jL+\ell }^{\left(T\right)}\left(r,n_{t}\right),E_{jL+\ell }^{\left(T\right)}\left(r,n_{t}\right)\right),\;j\in \mathbb{Z}\right\}$$
    are independent but have different laws.
3. We first note that $\left\{H_{k},\;k\in \right\}$ is an ergodic Gaussian process, which implies that it is also weakly mixing [35]. (See [36] for a definition of a weakly-mixing process.) Since $\left\{Z_{k},\;k\in \right\}$ is an i.i.d. Gaussian process and independent from $\left\{H_{k},\;k\in \right\}$, it follows from ([36], Proposition 1.6) that $\left\{\left(H_{k},Z_{k}\right),\;k\in \right\}$ is jointly ergodic.
  We next evaluate the process $\left\{\left({\hat{H}}_{k}^{\left(T\right)},H_{k},Z_{k}\right),\;k\in D\right\}$. Please note that this process cannot be expressed directly as a time-invariant function of $\left\{\left(H_{k},Z_{k}\right),\;k\in \right\}$. Indeed, by assuming k=jL+ℓ, we can see from (A5) that the function to produce ${\hat{H}}_{k}^{\left(T\right)}$ from $\left\{\left(H_{k},Z_{k}\right),\;k\in \right\}$ depends on the time index *k* via *ℓ*. To sidestep this problem, and to facilitate the analysis, we introduce a “dummy” matrix-valued process $\left\{A_{k,\ell },\;k\in \right\}$ of dimension $n_{r}\times n_{t}$, where the row-*r* column-*t* entry of $A_{k,\ell }$ is given by
  (A9) $$A_{k,\ell }\left(r,t\right)=\sum_{\tau =-T}^{T-1}\alpha_{-\tau L,\ell }\left(r,t\right)\left(\sqrt{\frac{\mathrm{SNR}}{n_{t}}}H_{k-\tau L-\ell +t-1}\left(r,t\right)+Z_{k-\tau L-\ell +t-1}\left(r\right)\right).$$
  Here the coefficients $\alpha_{-\tau L,\ell }$, τ=−T,⋯,T have the same value as those in (A5) for a given *L* and *ℓ*. Consequently, for every $\ell =n_{t},\cdots ,L-1$, the process $\left\{A_{k,\ell },\;k\in \mathbb{Z}\right\}$ is a time-invariant function of $\left\{\left(H_{k},Z_{k}\right),\;k\in \mathbb{Z}\right\}$ that coincides with ${\hat{H}}_{k}^{\left(T\right)}$ for k=jL+ℓ. This in turn implies that for every $\ell =n_{t},\cdots ,L-1$, the process $\left\{\left(A_{k,\ell },H_{k},Z_{k}\right),\;k\in \mathbb{Z}\right\}$ is jointly weakly mixing. Furthermore, by the definition of weakly mixing [35,36,37], the process $\left\{\left(A_{jL+\ell ,\ell },H_{jL+\ell },Z_{jL+\ell }\right),\;j\in \mathbb{Z}\right\}$ (for every $\ell =n_{t},\cdots ,L-1$) is also jointly weakly mixing. Since for k=jL+ℓ, k∈D, the matrix $A_{jL+\ell ,\ell }$ is identical to ${\hat{H}}_{jL+\ell }^{\left(T\right)}$, it follows that the process $\left\{\left({\hat{H}}_{jL+\ell }^{\left(T\right)},H_{jL+\ell },Z_{jL+\ell }\right),\;j\in \mathbb{Z}\right\}$ (for every $\ell =n_{t},\cdots ,L-1$) is jointly weakly mixing, which implies ergodicity.
  We finally evaluate the joint behavior of the two processes $\left\{\left({\hat{H}}_{jL+\ell }^{\left(T\right)},H_{jL+\ell },Z_{jL+\ell }\right),\;j\in \mathbb{Z}\right\}$ and $\left\{X_{jL+\ell },\;j\in \mathbb{Z}\right\}$ for $\ell =n_{t},\cdots ,L-1$. Since, for every $\ell =n_{t},\cdots ,L-1$, the process $\left\{X_{jL+\ell },\;j\in \mathbb{Z}\right\}$ is i.i.d. and independent from $\left\{\left({\hat{H}}_{jL+\ell }^{\left(T\right)},H_{jL+\ell },Z_{jL+\ell }\right),\;j\in \mathbb{Z}\right\}$, we have by ([38], Lemma 2) that
  (A10) $$\left\{\left({\hat{H}}_{jL+\ell }^{\left(T\right)},\;H_{jL+\ell },\;Z_{jL+\ell },\;X_{jL+\ell }\right),\;j\in \mathbb{Z}\right\},\;\ell =n_{t},\cdots ,L-1$$
  is jointly ergodic.
4. Please note that the process $\left\{{\hat{H}}_{k}^{\left(T\right)},\;k\in D\right\}$ is a function of $\left\{\left(H_{k},Z_{k}\right),k\in P\right\}$. Since $\left\{Z_{k},\;k\in D\right\}$ has zero mean and is independent from $\left\{\left(H_{k},Z_{k}\right),\;k\in P\right\}$ and $\left\{X_{k},\;k\in D\right\}$, it follows that for every of $\ell =n_{t},\cdots ,L-1$ (which correspond to k∈D),
  (A11) $$E\left[Z_{\ell }^{†}{\hat{H}}_{\ell }^{\left(T\right)}X_{\ell }\right]=0.$$

## Appendix B. Variance of the Interpolation Error for $L\leq \frac{1}{2\lambda_{D}}$

Recall that the variance of the interpolation error tends to the following value as *T* tends to infinity:

(A12) $$\begin{array}{c} \epsilon_{\ell }^{2}\left(t\right)=1-\int_{-1/2}^{1/2}\frac{\mathrm{SNR}{\left|f_{L,\ell -t+1}\left(\lambda \right)\right|}^{2}}{\mathrm{SNR}f_{L,0}\left(\lambda \right)+n_{t}}d\lambda \end{array}$$

where

(A13) $$f_{L,\ell }\left(\lambda \right)=\frac{1}{L}\sum_{\nu =0}^{L-1}{\bar{f}}_{H}\left(\frac{\lambda -\nu }{L}\right)e^{i2\pi \ell \frac{\lambda -\nu }{L}},\;-\frac{1}{2}\leq \lambda \leq \frac{1}{2}.$$

This can be lower-bounded as

$$\begin{array}{ccc} (A14) & \epsilon_{\ell }^{2}\left(t\right)= & \int_{-1/2}^{1/2}\frac{n_{t}f_{L,0}\left(\lambda \right)}{\mathrm{SNR}f_{L,0}\left(\lambda \right)+n_{t}}d\lambda +\int_{-1/2}^{1/2}\frac{\mathrm{SNR}\left({\left[f_{L,0}\left(\lambda \right)\right]}^{2}-{\left|f_{L,\ell -t+1}\left(\lambda \right)\right|}^{2}\right)}{\mathrm{SNR}f_{L,0}\left(\lambda \right)+n_{t}}d\lambda \\ (A15) & \geq & \int_{-1/2}^{1/2}\frac{\mathrm{SNR}\left({\left[f_{L,0}\left(\lambda \right)\right]}^{2}-{\left|f_{L,\ell -t+1}\left(\lambda \right)\right|}^{2}\right)}{\mathrm{SNR}f_{L,0}\left(\lambda \right)+n_{t}}d\lambda \end{array}$$

where the inequality follows because the first integral in (A14) is nonnegative. Defining $\ell^{\prime }≜\ell -t+1$, we next note that

$$\begin{array}{ccc} (A16) & {\left[f_{L,0}\left(\lambda \right)\right]}^{2}-{\left|f_{L,\ell^{\prime }}\left(\lambda \right)\right|}^{2} & =\frac{1}{L^{2}}\sum_{\nu =0}^{L-1}\sum_{\begin{array}{c} \nu^{\prime }=0,\\ \nu^{\prime }\neq \nu \end{array}}^{L-1}{\bar{f}}_{H}\left(\frac{\lambda -\nu }{L}\right){\bar{f}}_{H}\left(\frac{\lambda -\nu^{\prime }}{L}\right)\left[1-e^{i2\pi \ell^{\prime }\frac{\lambda -\nu }{L}}\cdot e^{-i2\pi \ell^{\prime }\frac{\lambda -\nu^{\prime }}{L}}\right]\\ (A17)\; & & =\frac{2}{L^{2}}\sum_{\nu =0}^{L-1}\sum_{\begin{array}{c} \nu^{\prime }>\nu \end{array}}^{L-1}{\bar{f}}_{H}\left(\frac{\lambda -\nu }{L}\right){\bar{f}}_{H}\left(\frac{\lambda -\nu^{\prime }}{L}\right)\left[1-\cos\left(2\pi \ell^{\prime }\frac{\nu^{\prime }-\nu }{L}\right)\right]. \end{array}$$

Since the summands are nonnegative, it follows that

(A18) $$\begin{array}{c} {\left[f_{L,0}\left(\lambda \right)\right]}^{2}-{\left|f_{L,\ell^{\prime }}\left(\lambda \right)\right|}^{2}\geq \frac{2}{L^{2}}{\bar{f}}_{H}\left(\frac{\lambda }{L}\right){\bar{f}}_{H}\left(\frac{\lambda -1}{L}\right)\left[1-\cos\left(\frac{2\pi \ell^{\prime }}{L}\right)\right]. \end{array}$$

The RHS of (A15) can thus be lower-bounded as

(A19) $$\begin{array}{cc} \int_{-1/2}^{1/2}\frac{\mathrm{SNR}\left({\left[f_{L,0}\left(\lambda \right)\right]}^{2}-{\left|f_{L,\ell^{\prime }}\left(\lambda \right)\right|}^{2}\right)}{\mathrm{SNR}f_{L,0}\left(\lambda \right)+n_{t}}d\lambda & \geq \frac{2\left[1-\cos\left(\frac{2\pi \ell^{\prime }}{L}\right)\right]}{L^{2}}\int_{L}\frac{\mathrm{SNR}\;{\bar{f}}_{H}\left(\frac{\lambda }{L}\right){\bar{f}}_{H}\left(\frac{\lambda -1}{L}\right)}{\mathrm{SNR}f_{L,0}\left(\lambda \right)+n_{t}}d\lambda \end{array}$$

where L denotes the interval in [−1/2,1/2] where ${\bar{f}}_{H}\left(\frac{\lambda }{L}\right)$ and ${\bar{f}}_{H}\left(\frac{\lambda -1}{L}\right)$ overlap.

We next express *L* as

(A20) $$L=\frac{1}{2\lambda_{D}}+\varepsilon$$

for some ε>0. Then, the interval L is of Lebesgue measure

(A21) $$\mu \left(L\right)=\min\left(1,2\lambda_{D}\varepsilon \right).$$

By Fatou’s lemma [39], we obtain that

$$\begin{array}{cc} \; & \underset{\mathrm{SNR}\to \infty }{lim inf}\frac{2\left[1-\cos\left(\frac{2\pi \ell^{\prime }}{L}\right)\right]}{L^{2}}\int_{L}\frac{\mathrm{SNR}\;{\bar{f}}_{H}\left(\frac{\lambda }{L}\right){\bar{f}}_{H}\left(\frac{\lambda -1}{L}\right)}{\mathrm{SNR}f_{L,0}\left(\lambda \right)+n_{t}}d\lambda \\ (A22)\; & \;\geq \frac{2\left[1-\cos\left(\frac{2\pi \ell^{\prime }}{L}\right)\right]}{L^{2}}\int_{L}\underset{\mathrm{SNR}\to \infty }{lim inf}\frac{\mathrm{SNR}\;{\bar{f}}_{H}\left(\frac{\lambda }{L}\right){\bar{f}}_{H}\left(\frac{\lambda -1}{L}\right)}{\mathrm{SNR}f_{L,0}\left(\lambda \right)+n_{t}}d\lambda \\ (A23)\; & \;=\frac{2\left[1-\cos\left(\frac{2\pi \ell^{\prime }}{L}\right)\right]}{L^{2}}\int_{L}\frac{{\bar{f}}_{H}\left(\frac{\lambda }{L}\right){\bar{f}}_{H}\left(\frac{\lambda -1}{L}\right)}{f_{L,0}\left(\lambda \right)}d\lambda . \end{array}$$

Since L is of positive Lebesgue measure, and since the integrand on the RHS of (A23) is strictly positive, it follows that [40]

(A24) $$\int_{L}\frac{{\bar{f}}_{H}\left(\frac{\lambda }{L}\right){\bar{f}}_{H}\left(\frac{\lambda -1}{L}\right)}{f_{L,0}\left(\lambda \right)}d\lambda >0.$$

Furthermore, for $\ell^{\prime }=\ell -t+1$ and $\ell =n_{t},\cdots ,L-1$, we have

(A25) $$\cos\left(\frac{2\pi \ell^{\prime }}{L}\right)<1.$$

Consequently, combining (A25) and (A24) with (A23), (A19), and (A15), we obtain the desired result

(A26) $$\underset{\mathrm{SNR}\to \infty }{lim inf}\;\;\epsilon_{\ell }^{2}\left(t\right)>0.$$
